# Robust hybrid feature-driven on-the-fly mapping enables freehand 3D panoramic photoacoustic angiography

**DOI:** 10.1038/s41377-026-02401-7

**Published:** 2026-07-22

**Authors:** Haishu Xin, Erqi Wang, Rui Ma, Xin Chen, Yanshen Guo, Xinyue Huang, Fanjia Zeng, Yuanzheng Ma, Kaipeng Zhang, Ting Guo, Zhiyang Wang, Wuyu Zhang, Fei Yang, Yuqin Zhang, Sihua Yang

**Affiliations:** 1 MOE Key Laboratory of Laser Life Science & Institute of Laser Life Science, South China Normal University, Guangzhou, China; 2 Guangdong Provincial Key Laboratory of Laser Life Science, College of Biophotonics, School of Optoelectronic Science and Engineering, South China Normal University, Guangzhou, China; 3 Guangdong Basic Research Center of Excellence for Structure and Fundamental Interactions of Matter, South China Normal University, Guangzhou, China; 4 Department of Electronic Engineering, The Chinese University of Hong Kong, Hong Kong, China; 5 Orthopaedic Medical Research Center, The First Affiliated Hospital of Shantou University Medical College, Shantou, China; 6 Institute of Data and Information, Tsinghua Shenzhen International Graduate School, Tsinghua University, Shenzhen, China; 7 Guangdong Provincial Key Laboratory of Artificial Intelligence in Medical Image Analysis and Application, Guangdong Provincial People’s Hospital (Guangdong Academy of Medical Sciences), Southern Medical University, Guangzhou, China; 8 Department of Radiology, The Affiliated LiHuiLi Hospital of Ningbo University, Ningbo, China

**Keywords:** Biophotonics, Photoacoustics

## Abstract

The vascular network defines the perfusion boundaries that are fundamental to surgical resection and functional preservation. Photoacoustic angiography (PAA) offers stereoscopic visualization and quantification of microvascular anatomy and hemodynamics in vivo, but existing implementations remain constrained in field of view and imaging speed, limiting the intraoperative utility. Here, we introduce a real-time PAA tracking and mapping strategy that estimates the six degree-of-freedom motion of a handheld probe and reconstructs panoramic three-dimensional vascular maps for surgical guidance. By associating geometric and intensity-based hybrid vascular features to prevent degeneracy, the method achieves robust online mapping with 99.67% accuracy, even under dynamic freehand operation. In rat radical gastrectomy, the approach expanded the imaging boundary by 42.3-fold to 50.84 mm³ within 16.7 s, enabling panoramic microvascular visualization beyond the surgical field for preoperative planning, postoperative evaluation, and distal hemodynamic surveillance. This on-the-fly, freehand, and field-of-view–unconstrained PAA establishes a practical framework for vascular-focused surgical interventions, supporting precise and timely decision-making.

## Abstract (web summary)

Geometry–texture dual-driven SLAM enables 3D photoacoustic panoramic vascular topology reconstruction and hemodynamic assessment, providing a new imaging strategy for surgical vascular navigation.

## Graphical abstract

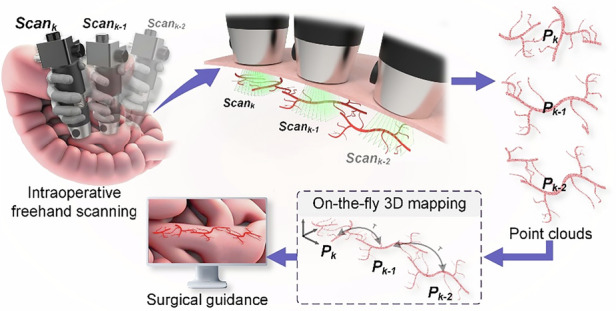

## Introduction

Detailed anatomical and functional characterization of blood vessels is essential for surgical interventions, with preoperative angiography delineating these features to guide surgical planning, preserve critical vessels, and evaluate vascular function. Photoacoustic angiography (PAA) integrates optical resolution with ultrasonic penetration, enabling highly sensitive label-free vascular angiography and emerging as a vital tool for surgical guidance and therapies performed by physicians or robots^1–5^. PAA provides lateral resolutions ranging from hundreds of nano-meters to tens of micro-meters, paving new avenues for intraoperative application^6–10^. PAA’s current trends toward high-speed^11–13^, miniaturization^14–17^, and handheld^18–20^capabilities exhibit potential in clinical applications^21–26^, such as neurovascular imaging for Alzheimer’s disease pathology progression^27–29^, oral vascular network imaging for health assessment^30^, and cerebral hemodynamic monitoring in stroke^31–36^. Photoacoustic molecular imaging can provide in vivo feedback during targeted therapy, enabling noninvasive monitoring of dynamic biological responses and further demonstrating the potential of photoacoustic imaging for deep-tissue dynamic tracking and quantitative functional analysis^37,38^. Meanwhile, recent advances in photoacoustic computed tomography (PACT) have highlighted the broader potential of photoacoustic imaging for noninvasive multi-organ visualization of anatomical structures and dynamic vascular changes in deep tissues, including the liver, brain, and heart^39–41^. However, PAA typically requires a trade-off between image resolution, imaging speed, and scanning degree-of-freedom(DOF)^42–45^. More fundamentally, this reflects an intrinsic systems-level constraint in photoacoustic angiography, in which improved spatial resolution is often accompanied by reduced instantaneous field of view and more stringent requirements on scanning stability and robustness. For example, handheld high-speed, high-resolution PAA devices designed for flexible operation often exhibit limited imaging field-of-view (FOV), because each single scan covers only a small scanning range, and a larger FOV can only be achieved by integrating multiple scanning ranges during freehand scanning. This presents challenges for application in surgical vascular guidance, as achieving on-site large-FOV volumetric imaging to cover complex vascular networks in various anatomical regions and obtaining real-time functional information are crucial^46,47^.

Currently, most freehand PAA systems rely on 2D image mosaicking to enlarge the FOV. These methods typically project 3D volumes into plane and align shared features to form a larger 2D image^17,48^, or further integrating simultaneous localization and mapping (SLAM) algorithm to achieve real-time tracking and FOV expansion in a 2D context^49^. However, such methods lose crucial depth information, failing to reconstruct non-planar vessel morphology and complex volumetric structures. Under non-rigid deformations like soft tissue shifting and respiratory motion, registration stability deteriorates due to accumulating inter-frame errors. This prevents the generation of true three-dimensional volumetric models, thereby limiting applications reliant on 3D data including vascular network modeling, anatomical structure identification, and path navigation. Furthermore, robustness is equally critical for in vivo handheld imaging. Medical imaging environments are often unstructured and highly dynamic, influenced by deformable soft tissue. Factors such as bleeding or blotches can also result in a lack of distinct texture features^50^. These challenges can significantly degrade the accuracy or lead to the failure of feature-based tracking methods. Adaptive excitation light-field control may improve photoacoustic imaging over curved organ surfaces by compensating for defocus and illumination nonuniformity. This enhances signal consistency and mapping robustness in complex in vivo environments^51–53^.

In this work, we present a handheld PAA system with a 532 nm wavelength laser, which is highly absorbent to hemoglobin. This system achieves a high imaging speed of 10 volumes/s within a scanning range of 2 × 1 mm, and simultaneously provides vector hemodynamic information of blood flow, enabling real-time visualization of flow velocity and direction. Then, we introduce photoacoustic angiography tracking and mapping system (PAATAM), a freehand 3D panoramic angiography that tracks the 6-DOF poses of the handheld scanner in real-time using 3D PAA images, enabling the construction of comprehensive 3D panoramic vascular maps without FOV constraints, offering on-site guidance. To address the challenges of estimation degeneracy in dynamic in vivo environments, we developed a robust hybrid feature-driven estimation strategy. PAATAM decouples raw data to independently extract geometric information related to vessel morphology and intensity information associated with absorption distribution, then integrates them into a sliding-window-based factor graph for tightly coupled estimation and mapping. Compared with SLAM methods^54^, PAATAM drives simultaneous localization and 3D mapping based on the geometric and intensity features of blood vessels. Through a cross-validation strategy of these two features, the 6-DoF spatial self-motion of the scanner is optimized and accumulated errors are suppressed to yield a globally consistent vascular map.

To comprehensively evaluate the performance of PAATAM, a series of experiments encompassing phantoms, live animals, and human subjects has been conducted. We benchmark accuracy of tracking and mapping methods through simulated scanning at different speeds, durations, and motion levels, validating its state-of-the-art performance, achieving in general relative error of less than 1%, and 99.7% accuracy even in high dynamics. Then, we validated the accuracy of blood flow velocity measurements through in vitro experiments. Finally, by implementing this handheld PAA panoramic vascular mapping in a rat’s gastrectomy, we expanded the imaging FOV from an initial volume of 1.2 mm^3^ to 50.84 mm^3^, which corresponds to a 42.37-fold increase within 16.77 s, demonstrating its usability. Moreover, we tested the intraoperative assessment of vessel functionality through blood flow velocity measurements. This on-the-fly, FOV-unconstrained, 3D PAA is potentially applicable in operating room settings, allowing surgeons to perform label-free vascular functional imaging of areas of interest. High-resolution imaging provides a clear anatomical understanding, enabling immediate surgical planning, decision-making, or treatment. Moreover, this work can be deployed on CPUs, and its performance does not rely on spatial positioning sensors. This aligns well with the trend towards miniaturization in photoacoustic imaging devices, allowing for seamless integration into other PAA research efforts and offering further possibilities for clinical applications. In the present study, PAATAM was evaluated retrospectively as a preclinical technical platform rather than being used to drive real-time surgical decisions, while its potential to support intraoperative planning and decision-making will be assessed in future surgeon-in-the-loop studies.

## Results

### Principle and implementation of PAATAM

**Fig. 1:**
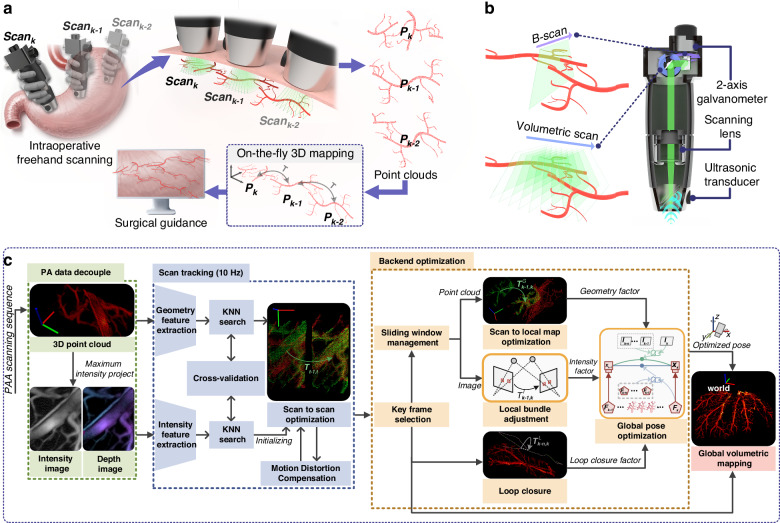
Illustration of the proposed PAATAM. **a** The pipeline for PAATAM. During intraoperative handheld scanning, photoacoustic signals are reconstructed into a 3D vascular map and displayed for surgical guidance in real time. **b** Schematic of the PAATAM. A focused laser excites ultrasonic waves that generate depth-resolved A-lines, scanning across tissue forms B-scans, and stacking B-scans yields volumetric images. **c** Principle diagram of the proposed robust hybrid-feature driven tracking and mapping

As illustrated in Fig. 1a, PAATAM aims to construct a 3D panoramic map in real-time from freehand scanning photoacoustic imaging. We designed a handheld optical-resolution photoacoustic angiography (OR-PAA) that employs a 160 kHz repetition rate, 532 nm wavelength laser, and utilizes a bi-axial galvanometer scanner for laser deflection (Fig. 1b). The size of the scanner (42 mm × 42 mm × 110 mm) makes it conveniently portable for handheld use. The high imaging speed of 10 volumes/s alleviates motion artifacts caused by involuntary motions such as heartbeat or breathing during imaging from live animals or humans (Supplementary Fig. 2). Then, a tracking and mapping system is applied to simultaneously estimate the six degrees of freedom of the scanner’s ego movement and obtain a globally consistent map (Fig. 1c). The raw photoacoustic data from real-time scanning are first reconstructed into a 3D point cloud, and also projected into 2D intensity and depth images.

During the scan tracking phase, the 3D point cloud and 2D images, respectively, provide vascular geometric features and intensity features related to absorption distribution. In feature association, a cross-validation strategy identifies and excludes outliers, mutually verifying the consistency of these two types of feature matches. Estimation based on intensity features provides an initial guess, which is further refined for frame-to-frame matching accuracy through estimation based on geometric features. Additionally, motion distortion correction is performed to ensure the scanner can move continuously rather than scan in a frame-by-frame stop motion manner. During the backend optimization phase, a factor graph is constructed within a sliding window for tightly coupled estimation and mapping. In the factor graph, joint optimization from scanning to map is performed, including constraints from geometric features, intensity features, and loop closure. Finally, the photoacoustic images are unified into consistent spatial coordinates based on the global scanner poses to construct the vascular map.

**Fig. 2:**
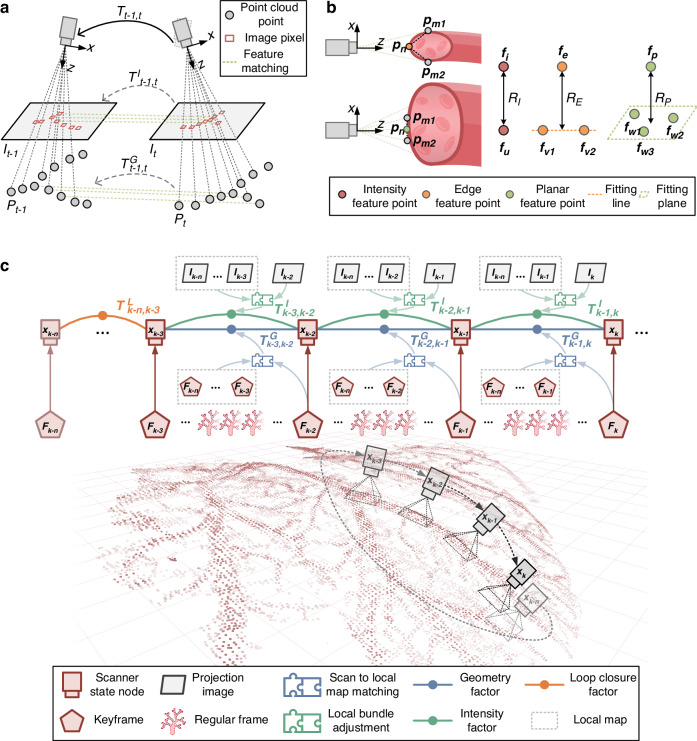
Demonstration of tracking and mapping. **a** The principle of simultaneous perception of vascular hybrid features. The front-end tracking module tracks intensity features to estimate the intensity-relative transformation$\,{T}_{t-1,t}^{I}$, and geometric features estimate the geometric-relative transformation ${T}_{t-1,t}^{G}$, thus obtaining the relative pose transformation ${T}_{t-1,t}$ of the scanner. **b** Geometric feature extraction method. Taking a 2D scanning cross-section as an example, local geometric coarseness is evaluated by the relative distance between each point and its neighbors. Points with higher coarseness are classified as edge feature points (${f}_{e}$), and those with lower roughness as planar feature points (${f}_{p}$). Edge feature points are matched with the two nearest points (${f}_{V1}$, ${f}_{V2}$) from the previous scan’s point cloud to form a line, generating the residual ${R}_{E}$. Planar feature points find the three nearest planar points (${f}_{W1}$, ${f}_{W2}$, ${f}_{W3}$) to fit a plane, resulting in the residual ${R}_{P}$. Selected intensity feature points (${f}_{i}$) on the projected image are matched with the most similar points (${f}_{u}$) of the visual descriptors from the previous scan, creating the point-to-point residual ${R}_{I}$. **c** Backend global optimization for scanner pose estimation and map construction. A factor graph is used to optimize the scanner state $x$, with all states at every moment jointly optimized in the sliding window to achieve a globally consistent mapping. The factor graph’s three types of constraints come from three computations: scan to local map optimization based on geometric features, local bundle adjustment based on intensity features, and loop closure

As depicted in Fig. 2a, we employ a lightweight, scan-matching-based front-end tracking module to iteratively estimate the relative transformation between two consecutive scans. The time delay of ultrasonic wave signals, caused by thermoelastic expansion following the absorption of light pulses by biological tissues, spatially delineates the geometric structure of blood vessels, with signal intensity indicating the absorption distribution. The scanner coordinate system is a three-dimensional coordinate system with the origin at the front of the scanner. Geometric features are directly extracted and matched in 3D space, whereas intensity features are processed as visual features in projected images. PAATAM simultaneously perceives both geometric and intensity features of photoacoustic imaging. This can improve feature point matching in complex or feature-sparse scenarios, offering effective constraints for estimation that notably ameliorate degeneracy issues. The problem of the tracking module is established through point-to-point, point-to-line, and point-to-plane feature matching, as shown in Fig. 2b. To avoid direct computation of huge point cloud data, we use a geometric feature extraction method similar to a previous method^55^. The edge features and planar features, respectively, represent smaller blood vessels or prominent edges within blood vessels and larger, flatter vascular surfaces.

Figure 2c illustrates the deployment of a sliding-window-based tightly coupled estimation and mapping on the backend, aimed at the global optimization of scanner pose estimation and the construction of a globally consistent map. Compared to loosely coupled fusion strategies, tight coupling considers intrinsic constraints among various observations, thus providing higher estimation precision. Furthermore, global optimization effectively reduces cumulative errors in long-term tracking. To ensure the real-time performance of the algorithm, the sliding window dynamically deletes old states and marginalizes them while adding new states. Additionally, we utilize a keyframe strategy to lower computational frequency. For frames where state changes exceed a predefined threshold, it is selected as the ${k}^{{th}}$ keyframe, ${F}^{k}$, and a new node ${x}^{k}$ representing the scanner’s pose is created in the factor graph.

**Fig. 3:**
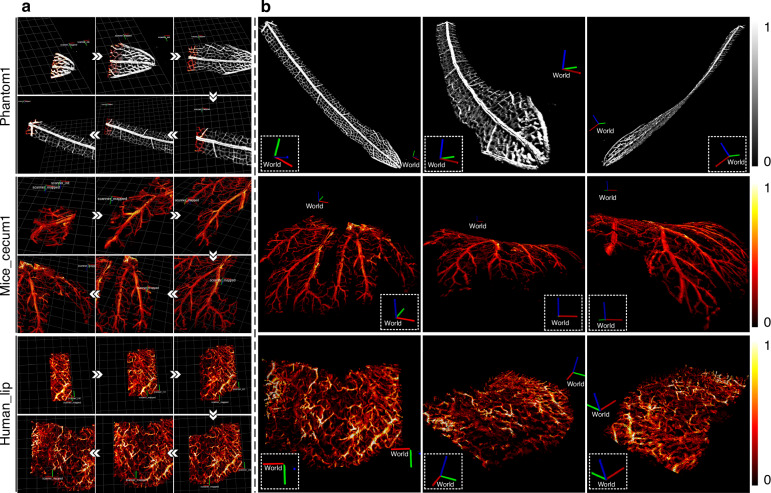
Examples of mapping processes and results from the phantom model, in vivo animal subject, and human subject. **a** The mapping process is illustrated by sequential snapshots. Arrows indicating the chronological order of the process. **b** Mapping results from different points of view. The white dashed box displays the orientation of the world coordinate system as seen from the current viewing perspective

To comprehensively assess the performance of PAATAM, we conducted handheld PAA experiments across phantoms, live animals, and human subjects (Fig. 3). In the freehand scanning mode, images were stitched online based on the spatial motion of the scanner. Snapshots taken at different time points illustrate the mapping process, with arrows indicating the probe’s movement direction, whereby the imaging field of view was progressively expanded as the probe advanced (Fig. 3a). Figure 3b shows the mapping results from different viewing angles, by selecting observation perspectives, panoramic stereoscopic 3D images of the scanned region were obtained, enabling full-scale visualization of the tissue’s three-dimensional architecture. To further evaluate PAATAM across different experimental subjects, a series of handheld photoacoustic microscopy scanning and tracking-mapping experiments were performed, covering multiple levels from phantoms and live animals to humans. In total, twelve datasets were acquired using two acquisition modes: freehand scanning and stage-controlled simulated scanning (Supplementary Table 1).

### Accuracy benchmarking on phantoms

Higher intraoperative scanning speeds improve diagnostic efficiency and reduce surgical duration and patient risk, but may compromise mapping accuracy and challenge real-time computation. Conversely, cautious scanning or larger regions of interest prolong acquisition, and long-sequence mapping is prone to drift, with errors accumulating between estimated and actual trajectories. To evaluate PAATAM’s performance and real-time computation across different speeds and durations, phantoms resembling vascular structures (leaf skeletons) were fixed on micro-precision multi-axis stages and scanned along preset trajectories, with imaging and mapping illustrated in Supplementary Videos 1–3. The phantoms were immersed in 1% agar to mimic soft tissue, and 1225 volumetric frames were acquired: 700 images from the slower maximum movement speed of 0.3 mm/s over 18.3 mm, 350 images from a medium maximum speed of 0.5 mm/s over 18.2 mm, and 175 images from the fastest 1.0 mm/s over 17.0 mm.

**Fig. 4:**
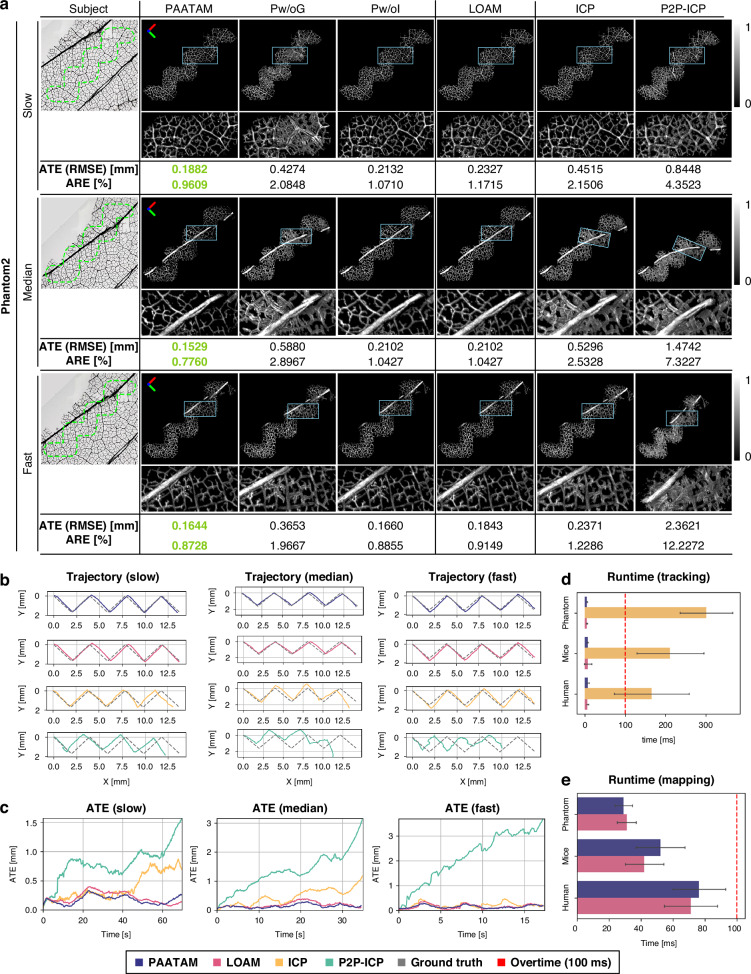
Accuracy benchmarking on phantoms. **a** Experimental results of phantom-based accuracy evaluation. Microscopic image of the phantom (far left) with the manually annotated scanning region outlined in green. The remaining panels show top-view 3D mapping results using PAATAM, PAATAM without geometry, PAATAM without intensity, LOAM, ICP, and P2P-ICP. World coordinate axes are indicated in the top-left corner; close-ups of wireframe regions are shown below. Quantitative comparison is based on ATE and RMSE across all timestamps, with the best result highlighted in green. Grayscale values denote normalized intensity. **b** Comparison of different estimated trajectories with the ground truth trajectory. The overlap with the ground truth trajectory displays prediction accuracy. **c** Changes in ATE over time for different estimated trajectories. ATE increases over time when prediction trajectories experience drift. **d**, **e** Average runtime statistics and standard deviation of different methods, with the red dashed line representing the 100 ms scanning interval

Experimental results are shown in Fig. 4. We compared PAATAM with widely used state-of-the-art algorithms, specifically choosing low-drift odometry and mapping (LOAM)^55^, the standard 3D model registration method Iterative Closest Point (ICP)^56^, and its variants point-to-plane ICP (P2P-ICP)^57^, and Generalized-ICP (G-ICP)^58^as baselines. Absolute trajectory error (ATE) and average relative error (ARE) were used to evaluate the tracking and mapping performance. Here, error denotes the deviation between the estimated trajectory and the ground truth, whereas accuracy is presented as a more intuitive percentage representation of the relative error. It should be noted that different methods have varying standards for selecting frames for mapping. Therefore, when evaluating trajectories, we first synchronize each trajectory with the ground truth trajectory based on time stamps, then use a rotational transformation to align them. It’s important to mention that ICP and its variants often do not achieve real-time performance. For comparison, trajectory time stamps were manually synchronized with the scanning time. PAATAM demonstrated the best performance at all three sequences with ATE root mean square errors (RMSE) of 0.1882 mm, 0.1529 mm, and 0.1644 mm, respectively (Fig. 4a), and achieved in general a relative error of less than 1%.

Additionally, in the comparison between PAATAM and Pw/oG, Pw/oI, we found that PAATAM using only geometric features as constraints performed similarly to LOAM. However, intensity features provided supplemental information in cases where geometric features degraded, enhancing the plausibility of estimation results. This demonstrates that the cooperation of both types of features improves performance. Under low-speed movement conditions (maximum speed of 0.3 mm/s), PAATAM, LOAM, and ICP all provided reasonable trajectory estimations (Fig. 4b). As the speed increased to 0.5 mm/s and 1.0 mm/s, the estimated trajectories of PAATAM and LOAM almost coincided with the actual trajectory, while ICP and P2P-ICP exhibited significant errors. We speculate that, under high-speed movement conditions, motion distortion caused by non-synchronous data acquisition significantly impacts registration accuracy. Hence, the robustness of PAATAM and LOAM can be attributed to their distortion correction mechanisms. Regarding cumulative errors or drift issues, we found that PAATAM and LOAM maintained lower drift even in longer data sequences. In contrast, ICP showed significant drift in a long sequence of 700 frames and reduced drift in medium and short sequences of 350 and 175 frames (Fig. 4c). This is attributed to ICP’s reliance on local registration from scan to scan, which easily accumulates errors over long sequences. In contrast, PAATAM and LOAM achieved more globally consistent results through global registration from scan to map in backend optimization.

The average runtime of PAATAM across all test data was below 80 ms at a scanning rate of 10 volumes/s (Fig. 4d), confirming real-time performance. Ablation experiments on five simulated sequences (Supplementary Table 2) yielded mean relative trajectory error(RTE) and ATE of 0.0189 ± 0.0129 mm and 0.1313 ± 0.0754 mm, respectively, demonstrating high precision. Integration of tracking, scan-to-local map optimization, and local bundle adjustment reduced ATE, improving global consistency despite a slight increase in RTE.

### 2.3 Advanced robustness in motion

Reliable 3D mapping generally depends on structured environments with stable features, yet continuous microscopic imaging of live subjects is challenged by deformable tissues, physiological motion, and handheld tremors, which introduce time-varying geometry and lead to the degeneracy or even invalidate optimization-based state estimation. To assess PAATAM’s robustness, Phantom3 was mounted on a motorized platform programmed to generate two rapid periodic displacements (0.064–0.64 mm, 2.5 Hz), simulating motion from respiration, heartbeat, or handheld operation.

**Fig. 5:**
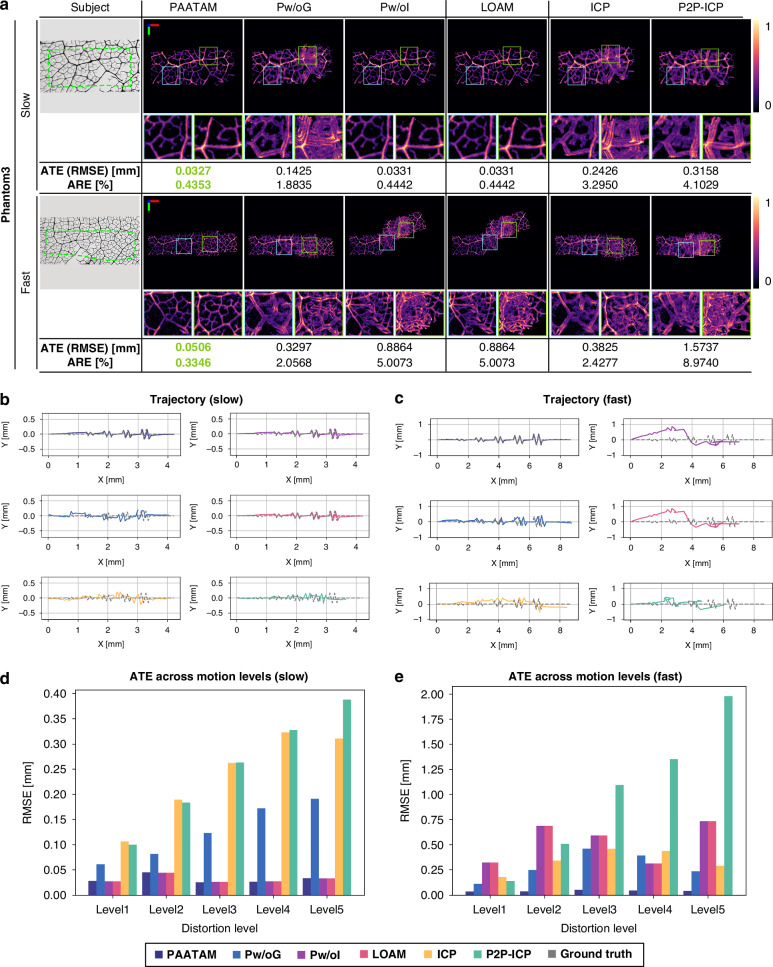
Metrics in simulated motion conditions. **a** Experimental results of phantom-based robustness evaluation. Microscopic image of the phantom (far left) with the manually annotated scanning region (green dashed line). Remaining panels show top-view volumetric mapping by PAATAM, PAATAM without geometry, PAATAM without intensity, LOAM, ICP, and P2P-ICP. World coordinate axes are shown in the top-left; wireframe close-ups are below. Quantitative comparison is based on ATE and RMSE across timestamps, with the best results highlighted in green. Grayscale denotes normalized intensity. **b**, **c** Comparison of different estimated trajectories with the ground truth trajectory. The slow sequence is set with a smaller movement speed (0.3 mm/s) and amplitude, while the fast sequence is set with a larger movement speed (1.0 mm/s).It should be noted that when the sample is in motion, the trajectory does not accurately represent the motion trajectory of the scanner. **d**, **e** Performance of different methods under varying levels of motion, with error calculated by computing the RMSE of ATE during motion

Figure 5 displays the experimental results, with Supplementary Video 4 and 5 providing a more intuitive understanding of the process. In addition to comparison with baseline methods, we also included PAATAM without geometry feature (Pw/oG) and PAATAM without intensity feature (Pw/oI) in the analysis to investigate the contributions of these two features to PAATAM’s performance. As shown in Fig. 5b, c, PAATAM, LOAM, and Pw/oI exhibited robustness for relatively mild motion conditions. However, only PAATAM maintained reliable function for more intense motion throughout the process, with only 0.3346% relative error even in high dynamics. PAATAM using only intensity features performed second best to PAATAM, while other methods encountered more critical failures. Moreover, by calculating the RMSE of the estimated trajectory during each motion, PAATAM consistently maintained low error levels in each motion (Fig. 5d, e). Comparison of PAATAM, Pw/oG, and Pw/oI indicates that incorporating intensity features enhances robustness to temporary geometric degeneracy or failure, distinguishing PAATAM from other approaches.

Scan-matching methods are recognized for their high precision in 3D point cloud registration, but their application in handheld PAA is constrained by dependence on initial values and sensitivity to outliers. The former arises from the non-convex nature of the problem and the reliance on local iterative optimization, whereby convergence to suboptimal minima may occur, resulting in inaccurate estimations. Under significant motion, the increased distance between adjacent point clouds can cause convergence to a local optimum in the absence of a reasonable initial estimate. Sensitivity to outliers further limits scan-matching, as the search for correspondences across candidate points and global transformation fitting can be biased by mismatches. These outliers might originate from point cloud deformation, partial matching, and other factors^59^. Soft tissue motion and deformation reduce valid matches and increase outlier risk. Leveraging local gradient statistics, PAATAM’s intensity features enhance distinctiveness, enabling robust matching with less reliance on initial values and providing effective constraints even under geometric degeneracy. The iterative estimation is shown in Supplementary Fig. 3 and Video 6.

### Freehand scanning performance across multiple subjects

**Fig. 6:**
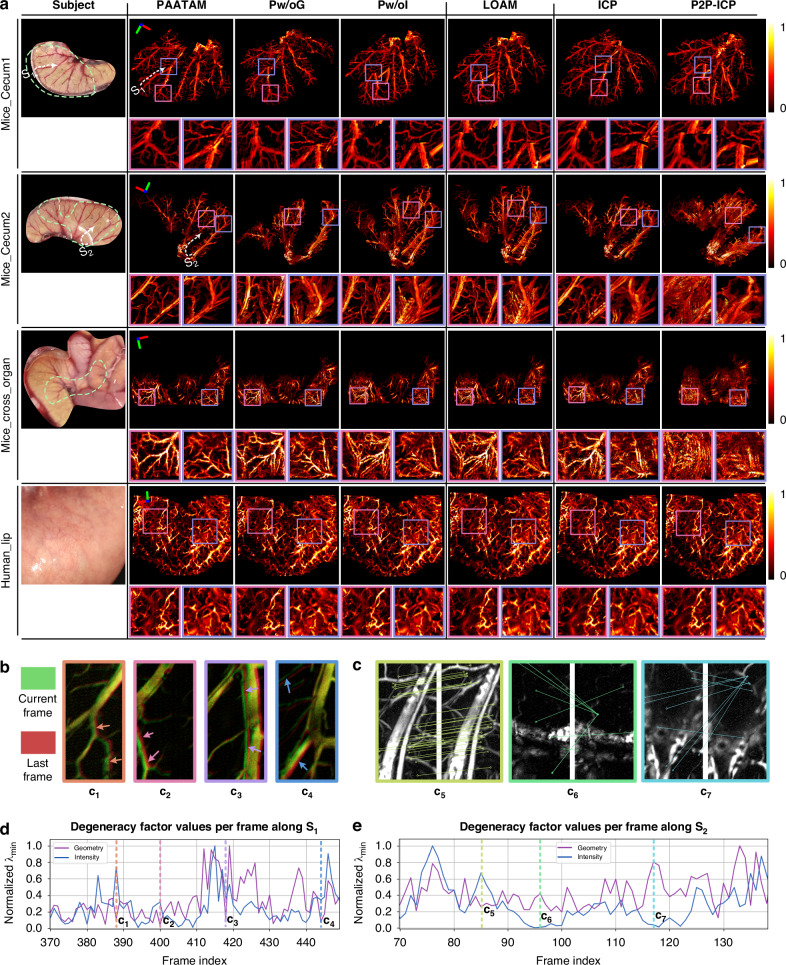
Freehand scanning performance across multiple subjects. **a** Handheld scanning results of different regions in vivo. Microscopic image of the specimen (far left) with the annotated scanning area (green dashed line). Remaining panels: top-view volumetric mapping by PAATAM, PAATAM without vascular structure feature, PAATAM without tissue texture feature, LOAM, ICP, and point-to-plane ICP. World coordinate axes are shown top-left; wireframe close-ups are at the bottom. **b** Close-up images C1, C2, C3, and C4 show several motions occurring in living tissue along sweeping trajectory S1. These are depicted by overlapping adjacent point cloud frames, with arrows indicating the direction. **c** Close-up images on sweeping trajectory S2 show intensity feature matching on the intensity projection images, with C5 as a standard reference match, and C6, C7 showing erroneous matches under low- texture conditions like bleeding. Dots and lines represent feature points and their matching connections, respectively. **d** The degeneracy factor for the sweeping trajectory S1, with values normalized. **e** The degeneracy factor for the sweeping trajectory S2, with values normalized

We evaluated PAATAM in live mice and human subjects. In mice, abdominal scans deliberately included larger superficial vessels visible under microscopy, serving as anatomical references to assess mapping accuracy without ground truth. Although handheld scanning inherently lacks reference trajectories, results in Fig. 6a show that PAATAM achieves higher precision in reconstructing complete vascular maps of living organs. Supplementary Videos 7–14 further illustrate the full tracking and mapping process under in vivo challenges, including cardiac and respiratory motion, bleeding, and signal attenuation from insufficient acoustic coupling. These factors can compromise accuracy and occasionally lead to reconstruction failure, yet PAATAM demonstrates robustness in capturing continuous vascular networks under such conditions.

The close-up images in Mice Cecum1, indicated by pink-purple wireframes, show a significant difference where all methods except PAATAM exhibited noticeable misalignments due to multiple motions occurring along sweeping trajectory S1(Fig. 6a). We calculated the degeneracy factor ${{\lambda }}_{\min }$ using the method from previous method^60^ and found that these motions caused significant degeneracy in optimization problem constrained by vascular geometry (Fig. 6d). In contrast, optimization problem constrained by vascular absorption intensity was unaffected. Additionally, unlike wide-field PAA achieved through two-dimensional raster scanning, PAATAM’s handheld scanning approach can mitigate defocus issues associated with limited depth-of-focus when imaging curved-shaped samples (Supplementary Fig. 4). In Mice Cecum2, sweeping trajectory S2 exhibited notable bleeding. Close-up images C6 and C7 in Fig. 6c, compared to the regular C5, show a drastic reduction in the number of intensity feature matches due to low texture conditions, with numerous false positives. This caused severe degeneracy in intensity optimization, while geometric optimization was unaffected (Fig. 6e). This further supports the flexibility and potential clinical utility of PAATAM in complex multi-organ imaging scenarios. While sensitivity analysis (Supplementary Fig. 10) demonstrates hybrid tracking remains robust down to 40% feature retention, this minimum density is not an absolute number; it relies heavily on specific anatomical structures and spatial feature distribution.

Furthermore, the flexibility of PAATAM enables handheld free scanning to construct three dimensional models in anatomical areas that are difficult to reach or in organs where traditional fixed scanning platforms are impractical. We specifically chose the oral cavity for a flexible examination experiment to validate its potential in clinical applications. During these experiments, PAATAM achieved effective imaging depths of approximately 870 µm in the oral vasculature of a healthy adult volunteer and 720 µm in the gastric vasculature of a rat, while enabling three-dimensional visualization of the corresponding volumetric microvascular networks from multiple perspectives (Supplementary Fig. 4). This grants PAATAM immense potential in human body detection and diagnosis, especially in identifying and assessing a range of oral mucosal diseases. For example, the vertically growing microvessels observed, known as intrapapillary capillary loops (IPCL), changes in their morphology and distribution can serve as critical criteria for distinguishing between benign and malignant oral lesions. Human experiments were restricted to imaging the lower lip of healthy volunteers, without inclusion of pathological lesions or assessment in deep targets. Accordingly, the human data should be interpreted as a proof-of-feasibility demonstration for panoramic superficial microvascular imaging rather than clinical validation. Extension to pathological tissues and deeper organs will require dedicated future studies, potentially leveraging longer excitation wavelengths and endoscopic implementations.

### Preoperative mapping and postoperative evaluation for partial gastrectomy

**Fig. 7:**
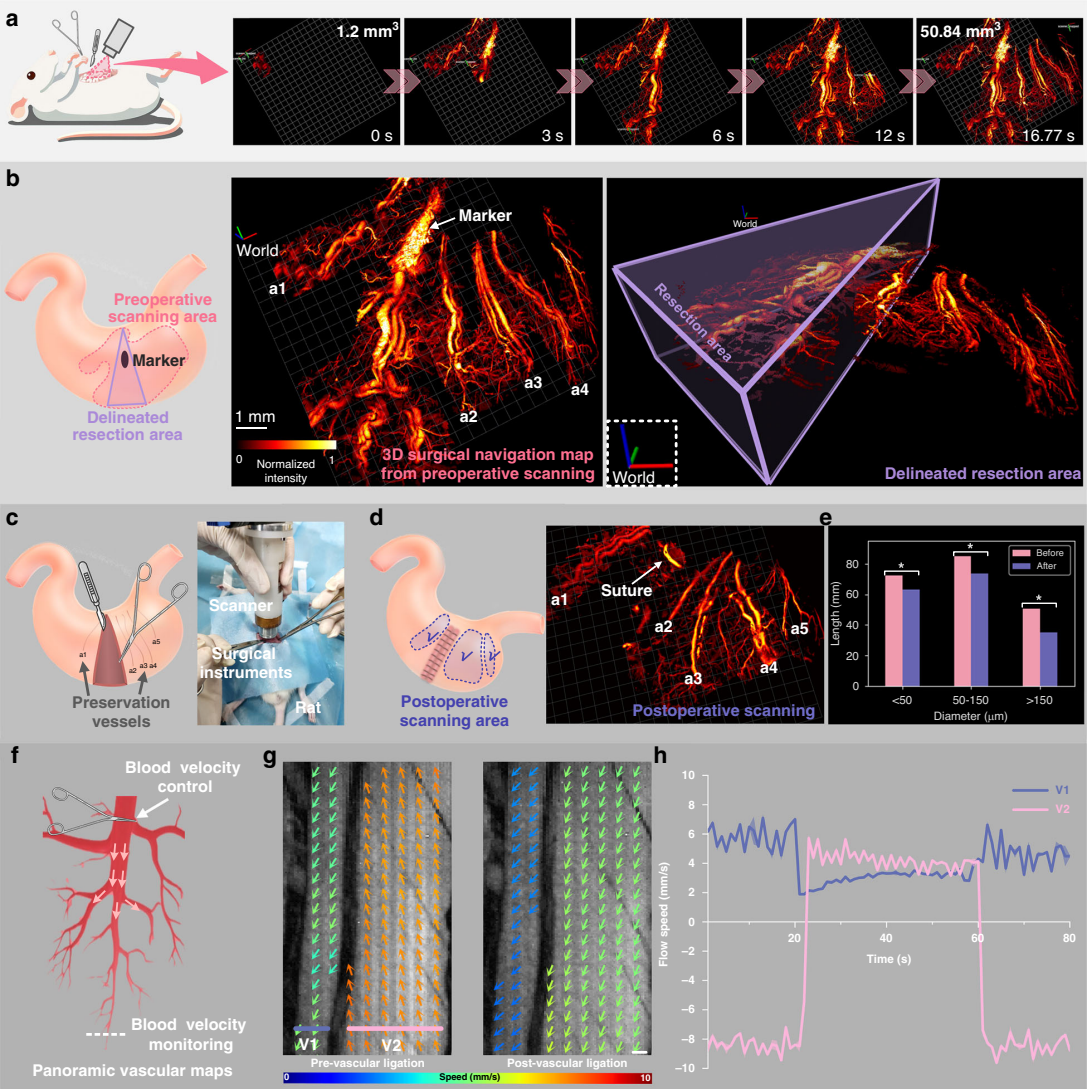
Preoperative mapping and intraoperative evaluation for the guidance of partial gastrectomy. **a** The process flow of PAATAM for intraoperative real-time mapping. **b** 3D mapping results of the scanning area, serving as a surgical navigation map. We used pigments that absorb 532 nm wavelength laser as markers to simulate a tumor. The figure on the right is a 3D surgical navigation map in another view. The resection area is marked by the surgeon. The surgical objective is to preserve other vessels outside the resection area. The white dashed box displays the orientation of the world coordinate system as seen from the current viewing perspective. **c** Schematic and experimental setup of the partial gastrectomy on rats. **d** Postoperative scanning of the preserved area. **e** The figure is the quantitative analysis of vessels before and after surgery (*P < 0.05). **f** Schematic diagram of the procedure for ligation of the left gastric artery and vein in rats. **g** Images of rat gastric vasculature before and after ligation. **h** Hemodynamic changes during left gastric vessel ligation in rat. Scale bar: 50 μm

Gastric cancer (GC) is the fifth most common malignancy globally and the fourth leading cause of cancer-related deaths^61^. Surgical intervention remains critical in GC treatment, with a shift towards function-preserving surgeries like proximal gastrectomy for early gastric cancer (EGC) to improve quality of life without compromising prognosis^62^. Recent studies highlight the importance of preserving gastric blood supply during gastrectomy to avoid severe complications. Additionally, in all surgical procedures, it is prudent to minimize damage to non-target blood vessels and reduce intraoperative bleeding whenever feasible^63^. PAATAM enables the construction of vascular networks before and after surgery without labeling. Surgeons can swiftly image the operative area using a handheld scanner, viewing high-resolution vascular images in real time on a monitor. In a partial gastrectomy in a rat (Fig. 7, Supplementary Video 15), we utilized PAATAM to construct a 3D vascular network of the left gastric artery (LGA) in the gastric surgical area. The imaging field expanded from an initial volume of 1.2 mm^3^ to 50.84 mm^3^, an increase of 42.37 times in 16.77 s (Fig. 7a). This 3D vascular network, serving as a surgical navigation map, illustrating the spatial relationship between the resection area and the preservation area, thus providing a basis for precise surgical planning (Fig. 7b). Following the partial gastrectomy based on preoperative planning, PAATAM is immediately used postoperatively to scan the preserved area (Fig. 7c, d). This allows for evaluating the preserved vessels, determining if the surgery achieved its intended effect, and providing postoperative treatment references. Figure 7e displays the quantitative analysis of the vasculature before and after surgery. Although the postoperative reduction in blood pressure resulted in a decrease in some blood vessels, the primary preserved arterial branches, designated as a1, a2, a3, a4, and a5, remained consistent in number. After partial gastrectomy, the total vessel length decreased significantly across all diameter groups (*P* < 0.05), with reductions of approximately 12.5% for vessels <50 μm, 14.1% for vessels 50–150 μm, and 30% for vessels >150 μm, indicating that the major vessels were effectively preserved after resection of the target area.

To validate the utility of photoacoustic optical flow method (PAOFM) for hemodynamic functional imaging, we employed this technique to achieve high-resolution visualization of gastric blood flow dynamics in living rats. Following acquisition of baseline vascular images, the left gastric artery and vein were subjected to ligation using hemostatic clamp, with subsequent clip release at 40 s post-ligation to capture hemodynamic data during the reperfusion phase. As illustrated in Fig. 7g, the pre-ligation vascular map (left panel) reveals opposing blood flow directions between the two target vessels (V1 and V2) alongside significant inter-vessel differences in flow velocity. In contrast, the post-ligation image (right panel) demonstrates a marked reduction in flow velocity across both vessels, accompanied by a reversal of flow direction in V2. This directional shift in V2 is attributed to the robust collateral circulation inherent to the venous system: occlusion of the primary gastric vein disrupts the canonical venous return pathway, prompting blood redistribution through intact collateral venous networks to maintain tissue perfusion. Fig. 7h quantifies vascular dynamics, with ligation performed at 20 s inducing sustained flow decline (V1: 73.3% vs V2: 66.5%) and clip removal at 60 s restoring velocities to baseline (Supplementary Video 16). These results demonstrate that PAOFM enables real-time, sensitive monitoring of vascular flow velocity and direction during ligation–reperfusion, underscoring its potential for early diagnosis and therapeutic evaluation of vascular disorders.

## Discussion

This study introduces photoacoustic microscopy tracking and mapping (PAATAM), a freehand 3D panoramic angiography to track the 6 degree-of-freedom handheld scanner poses in real-time via high-speed PAA images, and construct panoramic 3D vascular maps without field-of-view constraints, offering on-site guidance. This method enables freehand scanning while simultaneously constructing 3D panoramic vascular maps in real time. PAATAM demonstrated state-of-the-art performance, with an overall relative error of less than 1% and only 0.33% even under highly dynamic conditions. Although higher scanning speeds may introduce motion distortion and partial signal loss, particularly along the mechanical movement direction, the resulting degradation in registration accuracy remained limited, indicating the robustness of PAATAM under dynamic freehand scanning.

The PAATAM framework operates under a local rigid assumption, which is supported by our microscopic field of view and high volumetric imaging speed (10 Hz), ensuring that small areas behave almost rigidly between consecutive frames. Spatial variations predominantly arise from the scanner’s ego-motion rather than intrinsic local tissue warping. When intermittent, severe local distortions occur, the complement of the hybrid features prevents tracking failure caused unreliable constraint. However, continuous non-rigid soft tissue elasticity remains a challenge for long-term global map consistency. To definitively resolve cumulative deformation errors in future iterations, we plan to transition to deformable factor graphs. By utilizing the physical and functional tissue properties uniquely acquired by photoacoustic imaging to inform elastic regularization in the optimization backend, the system could actively account for tissue mechanics and maintain seamless global consistency.

PAATAM’s ability to rapidly image 3D anatomical structures offers a valuable tool for preoperative planning and postoperative evaluation, enhancing the precision of surgical interventions. Experiments were conducted on phantoms and living subjects, including mice and human oral cavities. Then, a partial gastrectomy in a rat was performed to demonstrate its capability to preserve key vascular structures. Furthermore, the flexibility and compatibility of PAATAM with existing handheld PAA devices and CPUs highlight its potential for widespread clinical adoption. Its absence of a requirement for spatial positioning sensors aligns well with the trend toward miniaturization in photoacoustic imaging devices.

PAATAM faces three main limitations. The first is the lack of consideration for tissue deformation during mapping. Complex deformations of soft tissues during scanning can reduce the accuracy of map construction. This limitation arises due to rigid assumptions. Although some motion distortion correction is implemented, it does not fully account for the deformation of vessels within soft tissues in photoacoustic imaging data. We plan to correct vascular geometric deformations in future work through sophisticated non-rigid registration and deformable optimization. The current system achieves real-time CPU performance below the 100 ms limit. Sliding-window marginalization maintains an O(1) graph optimization complexity, preventing latency growth during prolonged scans (Supplementary Fig. 11). Future hardware featuring higher scanning speeds will leverage GPU acceleration to sustain this on-the-fly capability.

The second limitation of the current prototype is limited optical depth of field. The optics are fixed to a superficial focal zone, and our experiments were designed to keep tissue curvature and axial motion largely within this range; when modest out-of-plane motion occurs, image quality primarily degrades through reduced local SNR rather than abrupt tracking failure. The current framework does not explicitly reject out-of-focus frames using SNR or contrast thresholds. Instead, low-quality data are suppressed implicitly through noise-threshold filtering, reduced valid feature extraction in defocused regions, and exclusion of unreliable constraints during backend optimization. Future work could incorporate explicit SNR or contrast-based frame rejection to further improve robustness. Future extensions to strongly curved or dynamically moving organs will require depth-robust solutions, such as extended–depth-of-field optics, dynamic focusing to improve defocus tolerance and enhance tracking and mapping robustness in complex organ geometries.

The third limitation lies in probe miniaturization, which places higher demands on the scanning strategy, optical energy delivery, and acoustic detection. MEMS-based scanning architectures may overcome the bulkiness and scanning nonlinearity associated with galvanometer systems^20^, while fiber-based laser delivery may further enhance transmission efficiency and flexibility^64^. Although the current system employs piezoelectric ultrasound detection, future designs may benefit from optical sensors, enabling more compact probe configurations and improved detection sensitivity^65^.

Additionally, the current PAATAM system is a compact handheld probe intended to be placed on or immediately adjacent to the target, which in preclinical settings preserved adequate perilesional workspace for instrument manipulation. A practical workflow would involve short scanning bouts performed by an assistant (or robotic holder) interleaved with surgical steps, with the reconstructed 3D map displayed on a dedicated monitor within the surgeon’s line of sight but outside the operative field to minimize distraction. Stable coupling in a bloody, dynamic environment would rely on a small-footprint, sheath-covered probe with acoustic gel and gentle constant pressure, while acknowledging that blood, motion, and crowding remain key translational challenges. Accordingly, future clinical versions will co-optimize probe footprint, working distance, holder design, and visualization strategy with surgeons. Integrating augmented or mixed reality technology can enhance the correlation between the photoacoustic surgical map and the anatomically visible sites during surgical guidance. This correlation could be achieved through optical or other sensing technologies and visualized using augmented reality to highlight anatomical structures or via mixed reality projection to provide images visible to the naked eye^66^.

This work is enabled by several advances that have only recently matured, including high-speed handheld optical-resolution photoacoustic imaging, real-time volumetric reconstruction, and hybrid-feature-driven factor-graph optimization for 6-DOF freehand tracking and mapping. Together, these developments make on-the-fly 3D panoramic photoacoustic angiography feasible beyond the conventional 2D mosaicking paradigm. The present work should be interpreted within the framework of an intrinsic systems-level trade-off in photoacoustic angiography: enhanced spatial resolution generally comes at the cost of a reduced instantaneous field of view, while simultaneously placing more stringent demands on scanning stability and system robustness. PAATAM does not eliminate these underlying constraints, but instead re-balances them by combining high-resolution local acquisition with real-time tracking and mapping, thereby substantially extending the effective 3D imaging volume. Future photoacoustic systems will therefore still require coordinated advances in optical design, scanning architecture, acoustic detection, and computational reconstruction, since improvements in one performance dimension are unlikely to be achieved without compromise in others. Several hurdles nevertheless remain for clinical translation, including limited depth of focus on curved organs, motion-related artifacts, and non-rigid tissue deformation, as well as the need for environmental robustness, sterile implementation, and standardized clinical workflows. Broader validation in pathological human tissues and prospective surgeon-in-the-loop studies will also be essential for clinical adoption.

## Materials and methods

### Design of handheld photoacoustic system

A pulsed laser (Mosquitoo X 532-2-V, InnoLas) was used, emitting a 532 nm wavelength laser at a frequency of 200 kHz. The laser was spatially filtered and coupled into a single-mode fiber with a core diameter of 4 µm using a fiber coupler (PAF2-7 A, Thorlabs). The beam was then collimated using a fiber port collimator (F240FC-532, Thorlabs) and reflected into a scanning lens (RMS-4X, Olympus) by a bi-axial galvanometer scanner (S-8107, Sunny Technology), focusing it onto the sample. The ultrasound waves generated from the sample absorbing these pulsed lasers were reflected by a 45-degree inclined 200 µm thick glass slide and detected by a cylindrical planar ultrasound transducer (15L1-6-SMA, Doppler Electronic Technologies Inc.). The transducer had an effective aperture of 4 mm. In the experiments, water was used as the acoustic coupling medium and was sealed within a transparent membrane inside the ultrasound detector. The PA signals received by the transducer were amplified by a 50 dB low-noise amplifier (LNA-650, RFBAY) and converted into digital signals by a high-speed data acquisition card (M4i.4450-x8, Spectrum) at a sampling rate of 250 MHz. The imaging range was set to 2 mm × 1 mm, with a volumetric imaging speed of 10 volumes/s (Supplementary Fig. 1, Fig. 5).

At 10 volumes/s, cardiac motion in rats (6–8 Hz) is not fully frozen; thus, heartbeat-related motion remains a non-negligible source of residual artifacts (Supplementary Fig. 6). In the current implementation, the method primarily mitigates respiratory motion and low-frequency drift, while partially reducing cardiac-induced blurring. Consecutive B-scans are stabilized using SIFT-based 2D registration (feature matching and homography warping), which improves in-plane vessel sharpness and attenuates small inter-frame displacements, but cannot fully correct for rapid pulsatile motion or through-plane deformation. Additional stability analysis showed that increasing the imaging frame rate substantially reduced inter-frame image jitter, with adjacent-frame misalignment decreasing from 8 pixels at 1 Hz to 2 pixels at 10 Hz, corresponding to an ~80% improvement in imaging stability (Supplementary Fig.7). This result further supports the advantage of high-frame-rate acquisition for suppressing motion-induced instability during in vivo scanning. To minimize the impact of residual cardiac motion on vessel diameter measurements, we averaged vessel diameters over multiple cardiac cycles. Additionally, vessel diameters were extracted through frame averaging and feature tracking across consecutive frames, ensuring that physiological changes in vessel size were distinguished from motion artifacts. Quantitative analysis of the 10 Hz imaging data showed that vessel diameter fluctuations were maintained within 5 pixels, with a relative jitter of under 5%(Supplementary Fig.8). Such minimal variance suggests that physiological motion artifacts primarily from cardiac and respiratory sources had a limited confounding effect on the accuracy of diameter measurements. We plan to integrate cardiac gating and deformable motion compensation in future iterations to address this limitation and improve the accuracy of vessel measurements.

Considering the distance for the sound waves reflected to the transducer, the data acquisition card was set to delay triggering to position the scanner coordinate system origin at the front of the scanner. To ensure real-time continuous sampling and data transfer, the acquisition card was configured in streaming mode, with onboard memory used for data buffering. After sampling each complete B-scan, an interrupt occurred, followed by data transfer via PCI Express x8 Gen 2. During the experiments, the acquisition and processing software ran on a 2.9 GHz eight-core CPU, with the operating system being the Robot Operating System (ROS) under Linux. Image reconstruction consumed three cores in total, with data processing, tracking, and mapping programs running on separate cores. Our software code and dataset are publicly available.

### Data decoupling and feature extraction

PAATAM simultaneously perceives the geometric and intensity features of photoacoustic imaging (Fig. 2a), with geometric features directly extracted and matched in three-dimensional space and intensity features processed as visual features in projected images. After imaging reconstruction at time $t$, a point cloud is generated, and points with intensity below the background noise are deleted. Clusters with less than 10 points are also removed, segmenting out the vascular point cloud ${P}_{t}$, where each point $p=\left\{x,y,z,i\right\}$ consists of its 3D coordinates in the scanner coordinate system and the photoacoustic signal intensity value. Geometric feature points are extracted from the raw point cloud to reduce computational load. Considering the axial resolution of PAA is relatively lower than the lateral resolution, we first retain the maximum value of all a-lines, i.e., deleting points that are not the maximum along the scanning axis to extract the most prominent features.

Let ${p}_{n}$ be a point on ${P}_{t}$, and M be a set of consecutive points ${p}_{n}$ on the same B-scan. Half of the points in *M* lie to the left of ${p}_{n}$ and half to the right of ${p}_{n}$. Using the 3D coordinates of points in the scanner coordinate system, the coarseness of the local geometric structure of ${p}_{n}$ in M can be evaluated

1 $$c=\frac{1}{|M|{{\cdot }}{||}{p}_{n}{||}}\left|\left|\sum _{m\in M,m\ne j}\left({p}_{n}-{p}_{m}\right)\right|\right|$$

Each B-scan is divided into two equal subregions for the more uniform selection of feature points, and points are sorted by $c$ within each subregion. Then, a threshold ${c}_{{th}}$ is used to differentiate feature types. Points with $c$ greater than ${c}_{{th}}$ form the edge feature set ${F}_{E}$, while those with $c$ less than ${c}_{{th}}$ form the planar feature set ${F}_{P}$. Next, visual features are acquired by projecting the point cloud into an intensity image. As shown in Fig. 2a, the point cloud is projected along the scanning axis for maximum value projection, obtaining the intensity image ${I}_{I}$ and corresponding depth image ${I}_{D}$. All pixel values are normalized to range between 0 and 255. Pixels without corresponding valid points are set to 0.

Then, features are extracted from ${I}_{I}$. Assuming significant motions from the scanner or live tissue during scanning, Scale-Invariant Feature Transform (SIFT) features are extracted. SIFT statistics gradients around a key point to form a descriptor, capturing local texture information of vessels. SIFT features have been applied in PAA registration tasks. They are highly distinctive, providing robust matches under significant distortions, 3D viewpoint changes, noise, and intensity variations. Then, these two-dimensional feature points are projected back into three-dimensional space, and their depth values are extracted from the depth image ${I}_{D}$. Using bilinear interpolation, we achieve sub-pixel accuracy in feature point depth. Thus, the intensity feature set ${F}_{I}$ and the corresponding set of SIFT feature descriptors $D$ are obtained.

### Lightweight front-end scan tracking

We utilized a lightweight front-end tracking module based on scan-matching, establishing optimization problems through point-to-point, point-to-line, and point-to-plane feature associations to estimate the relative transformation ${T}_{t-1,t}$ between scans at times$\,t-1$ and $t$ (Fig. 2a, b). The transformation matrix $T\in {SE}(3)$ is represented as$T=\left[R,d\right]$, where R∈SO(3) is a rotation matrix, and d∈${R}^{3}$ is a translation vector.

Intensity features, in contrast to geometric features, offer higher distinctiveness. They maintain good matching performance even in unstructured soft tissue environments experiencing geometric degeneracy. Hence, we optimize point-to-point distances of intensity features to estimate the initial transformation. Using the SIFT feature descriptor set *D*, the search for corresponding matches in the intensity feature sets ${F}_{t}^{I}$ at time $t$ within the previous set ${{F}}_{t-1}^{I}$ is conducted, resulting in matched feature sets ${{F}}_{t}^{I,m}$, $\,{{F}}_{t-1}^{I,m}$. For the intensity feature point ${f}_{i}$∈ ${{F}}_{t}^{I,m}$ and its matching point ${f}_{u}$ ∈ ${{F}}_{t-1}^{I,m}$ in the previous frame, the distance residual is calculated,

2 $${R}_{I}\left({f}_{i}\right)={w}_{i}{{||}{f}_{u}-{T}_{t-1,t}^{I}{f}_{i}{||}}^{2}$$

where ${w}_{i}$ is a weight based on L2 distance, and ${{T}}_{t-1,t}^{I}$ is the relative transformation optimized based on intensity features. $\,{{T}}_{t-1,t}^{I}$ is solved using the Levenberg-Marquardt algorithm and robust Huber cost function *ρ*,

3 $${T}_{t-1,t}^{I}=\arg \,\mathop{min}\limits_{{T}_{t-1,t}^{I}}\sum _{{f}_{i}\in {F}_{t}^{I,m}}\,\rho ({R}_{I}({f}_{i}))$$

Subsequently, we iteratively refine ${T}_{t-1,t}$ by geometric optimization involving point-to-edge and point-to-plane distance residuals. In each iteration, given edge feature points ${f}_{e}$∈ ${{F}}_{t}^{E}$, the geometric feature-based relative transformation ${T}_{t-1,t}^{G}$ (where the initial value is set to ${T}_{t-1,t}^{I}$), and transformed points $\widetilde{{f}_{e}}={T}_{t-1,t}^{G}{f}_{e}$, we search for the nearest points ${f}_{v1}$, ${f}_{v2}$ in ${{F}}_{E}^{t-1}$ to fit a line, and compute the distance residual,

4 $${R}_{E}\left(\,\widetilde{{f}_{e}}\right)=\frac{{\rm{||}}\left(\,\widetilde{{f}_{e}}-{f}_{v1}\right)\times \left(\,\widetilde{{f}_{e}}-{f}_{v2}\right){\rm{||}}}{{\rm{||}}{f}_{v1}-{f}_{v2}{\rm{||}}}$$

Similarly, for planar feature points ${f}_{p}$∈ ${{F}}_{P}^{t}$, we transform them to $\widetilde{{f}_{p}}$ = ${T}_{t-1,t}^{G}{f}_{p}$ and search for the nearest points ${f}_{w1}$, ${f}_{w2}$, and ${f}_{w3}$ in ${F}_{P}^{t-1}$ to form a plane, calculate the distance residual after excluding outliers,

5 $${R}_{P}\left(\,\widetilde{{f}_{p}}\right)={\left(\widetilde{{f}_{p}}-{f}_{w1}\right)}^{T}\cdot \left(\frac{\left({f}_{w1}-{f}_{w2}\right)\times \left({f}_{w1}-{f}_{w3}\right)}{{\rm{||}}\left({f}_{w1}-{f}_{w2}\right)\times \left({f}_{w1}-{f}_{w3}\right){\rm{||}}}\right)$$

The final relative transformation is obtained by optimizing using the L-M algorithm,

6 $${T}_{t-1,t}=\arg \,\mathop{min}\limits_{{T}_{t-1,t}^{G}}\mathop{\sum}\limits_{{f}_{e}\in {F}_{t}^{E}}\,\rho ({R}_{E}(\,\widetilde{f}_{e}))+\mathop{\sum}\limits_{{f}_{p}\in {F}_{t}^{P}}\rho ({R}_{P}(\,\widetilde{f}_{p}))$$

A cross-validation strategy is implemented to mitigate tracking instability from outlier feature matches. This approach leverages the unique information each feature type provides to detect outliers more accurately. For instance, a pair of points well-matched in intensity but with substantial geometric disparity is likely an outlier. We apply the random sample consensus method for intensity matches to estimate a homography transformation, using it to transform feature points and calculate geometric distances. A point far from its matched point is flagged as an outlier. In geometric matches, we identify outliers by calculating the intensity difference between matched points, discarding those exceeding a predefined threshold.

Additionally, we compensate for motion distortion in imaging data to facilitate freehand scanning. Since laser-scanning-based PAA acquires data non-simultaneously, movement induces distortion. Despite high imaging speeds of up to 10 volumes/s reducing most distortions, microscale errors can still lead to drift. We use ${T}_{t-1,t}$ to correct distortion in ${P}_{t}$. Assuming the motion is approximately uniform, ${T}_{t-1,t}$ is linearly interpolated,

7 $${T}_{s}=\frac{{t}_{s}-{t}_{0}}{{t}_{{scan}}}{T}_{t-1,t}$$

Here, ${t}_{s}$ represents the sampling time of point ${p}_{s}$ the sth point in point cloud ${P}_{t}.{t}_{0}$ is the scan’s start time, while$\,{t}_{{scan}}$, set to 0.1 s, denotes the total duration of a frame scan. This linear interpolation aims to reproject scanning points, gathered at varying times, back to their positions at the start of scanning.

### Sliding-window-based tightly coupled estimation and mapping

The backend performs global optimization for scanner pose estimation and map construction. Using a factor graph, we formulate this state estimation task as maximizing posterior probability. Assuming the data follows a Gaussian distribution, this can be simplified as solving a standard non-linear least squares problem. A keyframe strategy is employed to reduce computational frequency, selecting frames with state changes exceeding a threshold as the ${k}_{th}$ keyframe ${F}_{k}=\left\{{F}_{k}^{E},{F}_{k}^{P},{F}_{k}^{I}\right\}$ and creating a new node ${x}_{k}$ in the factor graph representing the scanner pose. Additionally, to ensure real-time algorithm performance, the sliding window dynamically discards and marginalizes old states, adding new states. The world coordinate system, a three-dimensional coordinate system, is oriented by rotating the initial scanner coordinate system 90 degrees about the x-axis. The factor graph estimates the pose of the scanner in the world coordinate system. Ultimately, the original point cloud data are transformed into the map, constructing a globally consistent vascular map. For efficiency and accuracy, our method, inspired by LIO-SAM^67^, uses relative transformations as constraints between two states ${x}_{k-1}$ and ${x}_{k}$ in the factor graph, with geometric factor ${T}_{k-1,k}^{G}$, intensity factor ${T}_{k-1,k}^{I}$, and loop closure factor ${T}^{L}$(Fig. 2c). Loop closure detection is used to eliminate errors further, added as a constraint to the factor graph. Additionally, an option is used to decide whether to remove covered areas when constructing the map to update vascular dynamics.

Utilizing the preliminary pose estimation from the front-end scan tracking, the geometric features of the ${k}^{th}$ keyframe, ${F}_{k}^{G}=\left\{{F}_{k}^{E},{F}_{k}^{P}\right\}$, are transformed into the world coordinate system and matched with a local geometry feature map for scan to local map optimization. The nearest keyframes to ${F}_{k}^{G}$ within a radius of 1 mm are searched, forming the local geometric feature map ${M}_{k}^{G}=\left\{{M}_{k}^{E},{M}_{k}^{P}\right\}$. To avoid stacking repetitive feature points, we voxel down sample ${M}_{k}^{G}$ with a resolution set to the PAA resolution. Feature extraction for ${M}_{k}^{G}$ can be achieved through Principal Component Analysis (PCA). For each point in ${M}_{k}^{G}$, the nearest 5 points are searched to form a set N, and calculate the covariance of N along with its eigenvalues and eigenvectors. If there is one larger eigenvalue and two smaller ones, the point belongs to${M}_{k}^{E}$ ; if there are two larger and one smaller eigenvalue, the point belongs to ${M}_{k}^{P}$. Let ${T}_{k}^{G}$ be the pose of ${F}_{k}^{G}$ in the world coordinate system. Following the geometric optimization method in scan tracking, the distance between ${F}_{k}^{G}$ and ${M}_{k}^{G}$ in the world coordinate system is minimized to estimate ${T}_{k}^{G}$. The geometric factor, a relative transformation, is added as a constraint between ${x}_{k-1}$ and ${x}_{k}$ in the factor graph,

8 $${T}_{k-1,k}^{G}={\left({T}_{k-1}^{G}\right)}^{T}{T}_{k}^{G}$$

Similar to the local bundle adjustment (BA) strategy in vision-based SLAM, a local visual sliding window $W$ is employed, encompassing past keyframes that share the view with the$\,{k}^{th}$ keyframe. Considering the possibility of scanning revisiting historical locations in the future, we opt to search for keyframes within a certain distance to construct $W$, rather than within a specific length of time. This approach allows the sliding window to cover some temporally distant but spatially close frames. Using the intensity feature descriptors $D$, ${F}_{K}^{I}$ is matched with $W.\,\,J$ defined as the set of matched points between ${F}_{K}^{I}$ and the ${w}^{th}$ keyframe in $W$. Let ${T}_{K}^{I}$ be the pose of ${F}_{K}^{I}$ in the world coordinate system. The pose ${T}_{K}^{I}$ can be obtained by minimizing the distance residuals in the world coordinate system between keyframe points ${f}_{j}$ and their matching points ${f}_{j}^{{\prime} }$ in $W$,

9 $${T}_{k}^{I}={{arg}}\,\mathop{{{min}}}\limits_{{T}_{k}^{I}}\mathop{\sum}\limits_{\omega \in W}\,\mathop{\sum}\limits_{j\in J}\rho (\parallel {T}_{k}^{I}{f}_{j}-{T}_{w}{f}_{j}^{{{{\prime} }}}{\parallel }^{2})$$

where ${T}_{w}$ is the transformation matrix of the ${w}^{{th}}$ keyframe in the sliding window in the world coordinate system, calculated from previous factor graph optimization. The intensity factor is a relative transformation, added as a constraint between ${x}_{k-1}$ and ${x}_{k}$ in the factor graph,

10 $${T}_{k-1,k}^{I}={\left({T}_{k-1}^{I}\right)}^{T}{T}_{k}^{I}$$

Although constraints derived from vascular hybrid-feature enhance the precision and robustness of the estimation, there may still be extreme cases where one type of factor completely fails. In such scenarios, we aim to prevent the introduction of these factors as constraints into the factor graph. We use the method^60^ from for failure detection, calculating the degeneracy factor in the first iteration of optimization. When the degeneracy factor falls below a certain threshold, it is considered a failure. For nonlinear optimization in state estimation, iterative solutions to least squares problems can be used,

11 $${arg }\,\mathop{{min }}\limits_{x}\parallel {Ax}-b{\parallel }^{2}$$

The degeneracy factor is defined as:

12 $$D={{\rm{\lambda }}}_{\min }+1$$

where ${{\lambda }}_{\min }$ is the smallest eigenvalue of ${A}^{T}A$. When $D$ is below the threshold, it is judged as a failure, and the constraint will not be added to the factor graph.

### Animals preparation

We used BALB/c mice (female; body weight: 20–30 g) and SD rats (female; body weight: 200–250 g) for in vivo studies. Mice were housed according to standard regulations and had free access to food and water during a 12-h light/dark cycle. BALB/c mice were anesthetized with 3% sodium pentobarbital (3 g/100 ml) by intraperitoneal injection and kept immobile for internal organ imaging. The animals’ respiration and body temperature were continuously monitored throughout the experiment. Ultrasound gel was used as a coupling medium between the tissue surface and the scanner to facilitate ultrasound signal transmission. All animal procedures comply with the Guidelines for the Care and Use of Laboratory Animals issued by the National Institutes of Health (NIH).

### Gastrectomy in rats

Sprague–Dawley rats were anesthetized with isoflurane and positioned supine for gastrectomy and hemodynamic imaging experiments. Body temperature was maintained at 36.5 °C using a heating pad, and respiration and temperature were continuously monitored. After depilation and Iodine povidone disinfection of the abdominal region, a midline incision ( ~ 3 cm) was made under sterile draping to expose the stomach. For hemodynamic control, regions of interest on the gastric surface were selected, and the left gastric artery and vein were transiently occluded using tigergene microsurgical hemostatic clips. A pigment marker (3 mm × 0.75 mm) was placed on the greater curvature to simulate a tumor. Panoramic vascular mapping with PAATAM was then performed to guide the planned partial gastrectomy. The gastric wall was dissected sequentially with scissors under forceps assistance, with hemostasis achieved using vascular clips. Targeted gastric tissue was excised while preserving essential vessels, which were ligated with sutures. Anastomosis of the gastric wall was performed with interrupted sutures, followed by abdominal irrigation with saline. The surgical field was inspected for hemostasis and integrity before closure. A postoperative PAATAM scan was acquired to validate the conformity of the resection with preoperative planning.

### In vivo experiment of measuring blood flow velocity

To determine blood flow velocity in gastric vessels using the photoacoustic optical flow method (PAOFM), this study extracts and tracks red blood cells(RBCs) displacements from a continuous MIP image sequence and converts those displacements into calibrated velocity vectors. Specifically, within a user-defined ROI (vessel mask) an optical-flow algorithm is applied under the intensity-consistency assumption to compute per-pixel displacement vectors between consecutive frames, (Δx, Δy), which correspond to the same RBC-related pixels across frames^68^. To validate the accuracy of PAOFM for quantitative flow velocity imaging, we performed experiments using a PDMS microfluidic flow phantom. A syringe pump (KD Scientific, LEGATO 210) was used to drive flow through a microchannel with an inner diameter of 100 μm, filled with rat whole blood. Microfluidic calibration further demonstrated the quantitative reliability of the flow velocity measurements, with excellent agreement between the photoacoustic optical-flow results and the reference velocities (R² = 0.99973, K = 1.005) (Supplementary Fig. 9).

The resulting displacement fields for the x and y components are denoted ${\mathop{F}\limits^{ \sim }}_{{dx}}$ and ${\mathop{F}\limits^{ \sim }}_{{dy}}$. Each component is decomposed by singular-value decomposition into spatial and temporal modes,

13 $${\widetilde{F}}_{{dx}}={U}_{x\left(:,1:k\right)}\mathop{\Sigma }\limits_{x}\left({1:k,1:k}\right){V}_{x\left(:,1:k\right)}^{T}$$

14 $${\widetilde{F}}_{{dy}}={U}_{y\left(:,1:k\right)}\mathop{\Sigma }\limits_{y}\left({1:k,1:k}\right){V}_{y\left(:,1:k\right)}^{T}$$

and then truncated to retain only the most significant k singular components. The denoised displacement fields are reconstructed as ${\mathop{F}\limits^{ \sim }}_{{dx}},{\mathop{F}\limits^{ \sim }}_{{dy}}$, effectively suppressing noise and uncorrelated motions while preserving dominant flow structure. To reduce the influence of spurious vectors caused by noise or tracking errors, the speed magnitude of each corrected vector is computed,

15 $$S=\sqrt{{v}_{x}^{2}+{v}_{y}^{2}}$$

and the histogram of $S$ is used to find the modal speed ${S}_{{mode}}$. Vectors are marked as outliers when $|{S}_{i}-{S}_{{mode}}|/{S}_{{mode}} > \delta$ ; outlier magnitudes are then clamped into the interval $[{S}_{{mode}}\pm \eta {S}_{{mode}}]$. Finally, using the known inter-frame time interval Δt, the corrected per-frame displacements are converted into velocity components,

16 $${v}_{x}=\frac{\Delta x}{\Delta t},{v}_{y}=\frac{\Delta y}{\Delta t}$$

and the local speed magnitude

17 $${v}_{f}=\sqrt{{v}_{x}^{2}+{v}_{y}^{2}}$$

## Supplementary information

Supplementary Information

Accuracy test at slower speed movement in a simulated scan

Accuracy test at medium speed movement in a simulated scan

Accuracy test at higher speed movement in a simulated scan

Simulated scan under various motions

Simulated scan under various motions

Visualization of iterative estimation

Handheld scanning in mice cecum1

Display of mice cecum1 reconstruction result in 3D

Handheld scanning in mice cecum2

Display of mice cecum2 reconstruction result in 3D

Handheld scanning in mice cross organ

Display of mice cross organ reconstruction result in 3D

Handheld scanning in human lip

Display of human lip reconstruction result in 3D

Vascular panoramic mapping for partial gastrectomy

In vivo monitoring hemodynamic during left gastric vessel ligation in rat

## Data Availability

All data needed to evaluate the conclusions in the paper are present in the paper or the Supplementary Information. The code used in this study is available from the corresponding author upon reasonable request.
