# Supplementary material for: Robust hybrid feature-driven on-the-fly mapping enables freehand 3D panoramic photoacoustic angiography
Source: Light Sci Appl. 2026 Jul 22;15:328. doi: 10.1038/s41377-026-02401-7 (PMC13392144; doi:10.1038/s41377-026-02401-7)
Supplement: Supplementary file 1 — Supplementary Information [file 41377_2026_2401_MOESM1_ESM.pdf]

Supplementary Materials:

Robust hybrid feature-driven on-the-fly mapping enables freehand 3D panoramic photoacoustic angiography

Haishu Xin[1,2,3†], Erqi Wang[1,2,4†], Rui Ma[1,2], Xin Chen[1,2], Yanshen Guo[1,2], Xinyue Huang[1,2], Fanjia Zeng[5], Yuanzheng Ma[6], Kaipeng Zhang[1,2], Ting Guo[7], Zhiyang Wang[1,2], Wuyu Zhang[1,2], Fei Yang[1,2], Yuqin Zhang[8], Sihua Yang[1,2,3] *

[1]MOE Key Laboratory of Laser Life Science & Institute of Laser Life Science, South China Normal University, 510631 Guangzhou, China.
[2]Guangdong Provincial Key Laboratory of Laser Life Science, College of Biophotonics, School of Optoelectronic Science and Engineering, South China Normal University, 510631 Guangzhou, China.
[3]Guangdong Basic Research Center of Excellence for Structure and Fundamental Interactions of Matter, South China Normal University,510006 Guangzhou, China.
[4]Department of Electronic Engineering, The Chinese University of Hong Kong, Hong Kong 999077 SAR, China.
[5]Orthopaedic Medical Research Center, The First Affiliated Hospital of Shantou University Medical College, 515063 Shantou, China.
[6]Institute of Data and Information, Tsinghua Shenzhen International Graduate School, Tsinghua University, 518055 Shenzhen, China.
[7]Guangdong Provincial Key Laboratory of Artificial Intelligence in Medical Image Analysis and Application, Guangdong Provincial People's Hospital (Guangdong Academy of Medical Sciences), Southern Medical University, 510080 Guangzhou, China.
[8]Department of Radiology, The Affiliated LiHuiLi Hospital of Ningbo University, 315211 Ningbo, China.

Correspondence: Sihua Yang(yangsh@scnu.edu.cn)
These authors contributed equally: Haishu Xin, Erqi Wang.

This PDF file includes:

Figs. S1 to S11
Tables S1 to S2
Movies S1 to S16

Other Supplementary Materials for this manuscript include the following:

Movies S1 to S16

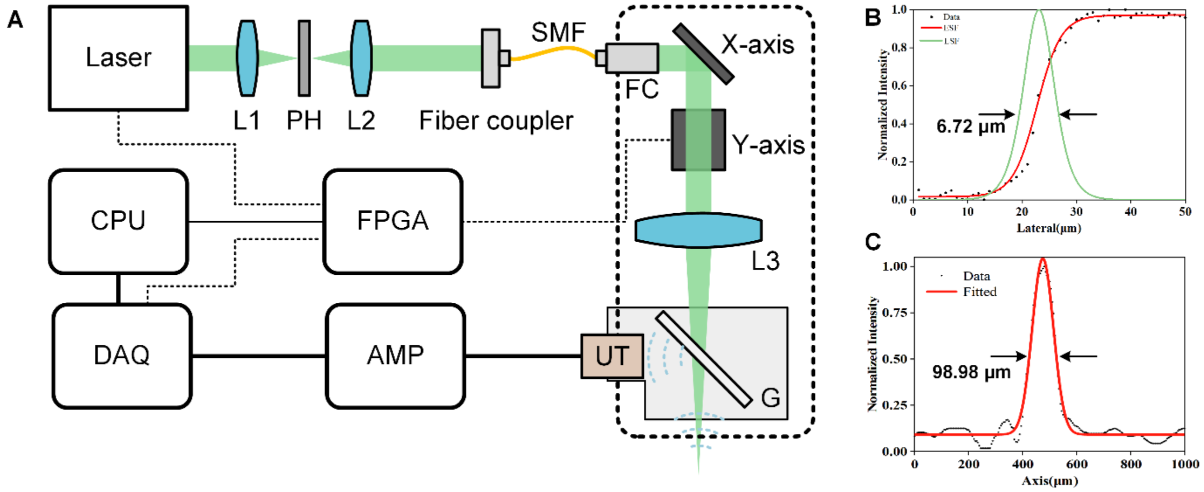

Fig. S1. System setup of the handheld PAA. A, The schematic of the PAA system. L1-L3: lens; PH: pinhole; SMF: single mode fiber; FC: fiber port collimator; X-axis: x-axis of galvanometer scanner; Y-axis: y-axis of galvanometer scanner; G: glass; UT: ultrasonic transducer; AMP: amplifier; DAQ: data acquisition card; FPGA: field-programmable gate array. **B**, Lateral Resolution: A surgical blade's edge was imaged, fitting the Edge Spread Function (ESF). The Line Spread Function (LSF) is derived, with a full width half maximum (FWHM) of 6.72 μm, determining lateral resolution. **C**, Axial Resolution: Imaging of carbon fibers, roughly 7 μm in diameter, yielded a Gaussian-fitted FWHM of 98.98 μm, determining axis resolution.

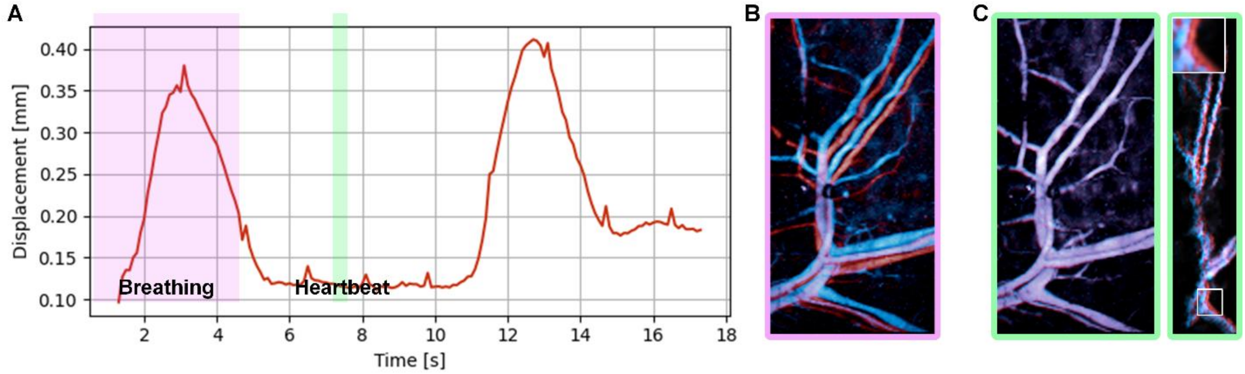

Fig. S2. PAATAM can stably image during respiration and heartbeats. A, Imaging captures large amplitude, low-frequency respiration, and small amplitude, high-frequency heartbeats, with displacements measured using PAATM. **B,** The respiration is marked in purple in A. **C,** The heartbeat is marked in light green in A, with the left image showing a top view and the right image a side view.

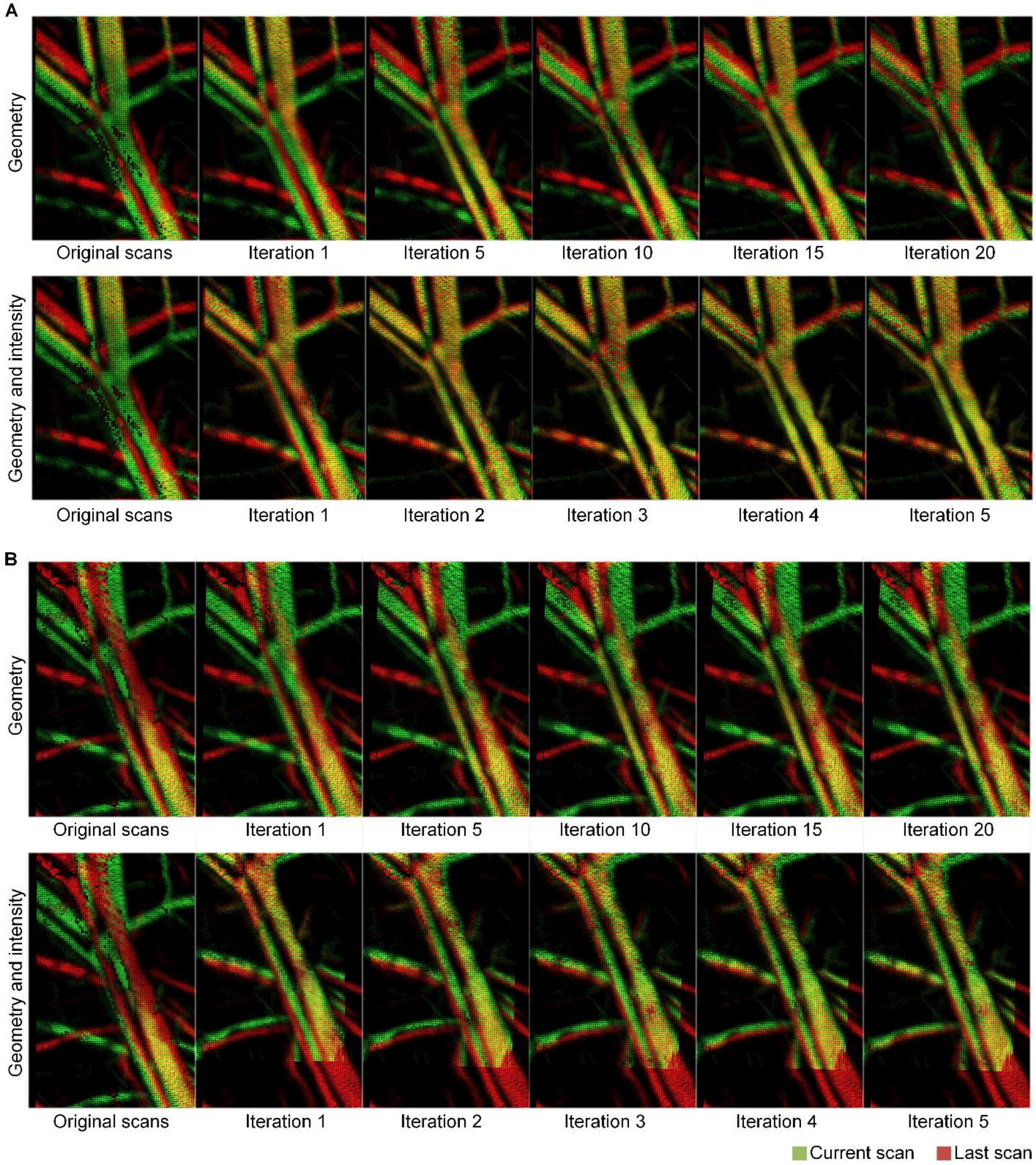

Fig. S3. Visualization of the iterative estimation. **A,** The first row shows the process of tracing solely the geometric features, requiring many iterations to converge. In contrast, the second row illustrates tracking both geometric and intensity features, achieving quick convergence. **B,** Solely tracking the geometric features results in a degeneracy in the dynamics, preventing convergence. Accurate convergence is ensured when tracking both features.

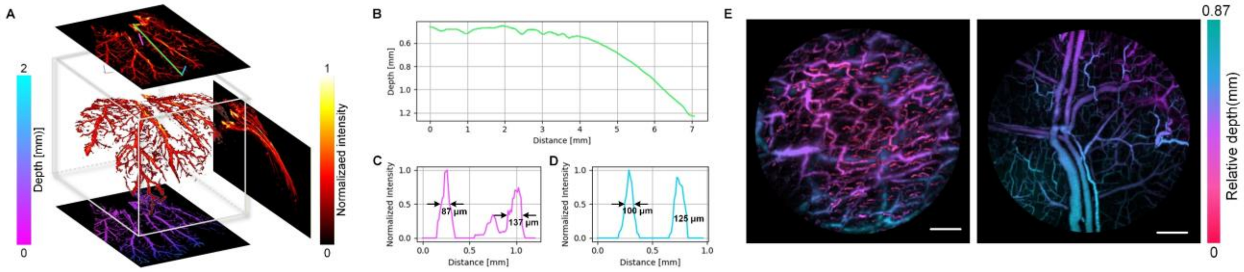

Fig. S4. PAATAM imaging of non-planar-shaped vascular morphology. A, Vascular imaging of a curved mouse organ, as an example, showcases point clouds, top view, side view, and depth images. **B,** The depth distribution along the green solid line in A illustrates the curvature of vascular morphology. **C,** Profile of smaller diameter vessels marked by magenta solid lines in A. **D,** Profile of two smaller diameter vessels marked by cyan solid lines in A. **E**, Left: PA depth-coded image of the oral microvasculature near the lower lip of a healthy volunteer, with an effective imaging depth of approximately 870 μm. Right: PA depth-coded image of the gastric microvasculature of a rat, with an effective imaging depth of approximately 720 μm. Scale bar, 500 μm.

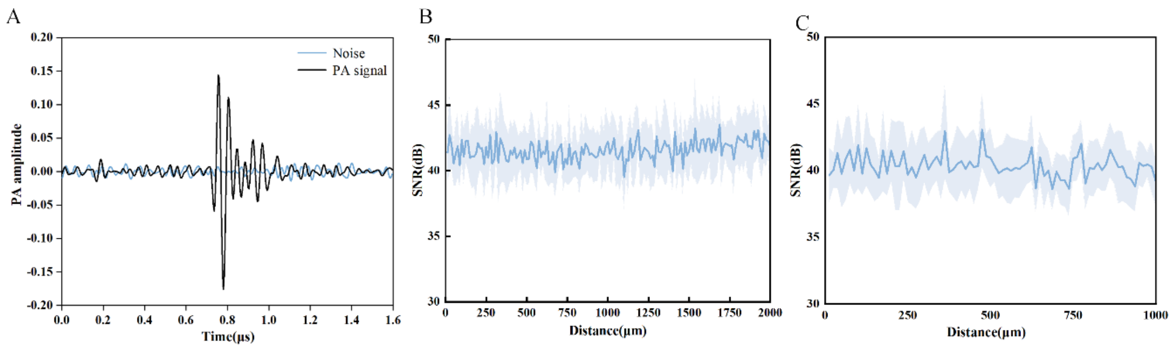

Fig. S5. SNR curves along the scanning direction. A, PA signal amplitude and the standard deviation of the noise; **B**, SNR was measured along the fast scanning direction; **C**, SNR was measured along the slow scanning direction.

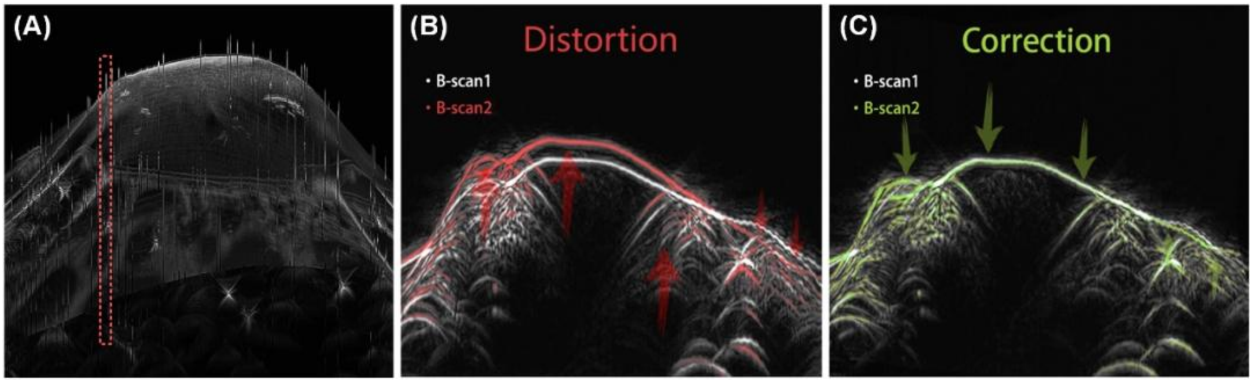

Fig. S6. Motion artifact correction based on rigid registration. **A**, Side view of a 3D photoacoustic image. The dashed lines the misregistration of the B-scans caused by the introduction of cardiac motion; **B**, Overlaid images of B-scans before correction; **C**, Overaid images of B-scans after correction.

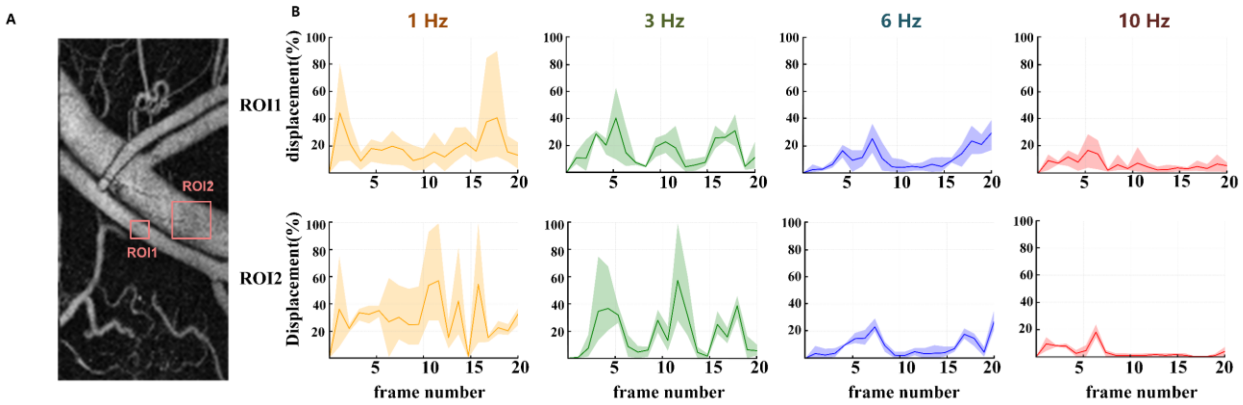

Fig.S7.Quantitative analysis of inter-frame jitter of two vascular ROIs at different frame rates. **A**, Maximum intensity projection images acquired at 10 Hz, indicating the two regions of interest (ROI1 and ROI2, pink boxes) for motion analysis. **B**, The temporal changes of non-rigid displacement between consecutive frames for ROI1 and ROI2 at four acquisition frame rates (1 Hz, 3 Hz, 6 Hz, 10 Hz). For each frame, the vascular skeleton within the ROI is divided into three equal segments along the vessel centerline, and the centroid displacement of each segment between adjacent frames is calculated. The solid line represents the mean displacement of the three segments, and the shaded area represents the range of the three segment displacements. The vertical axis shows inter-frame displacement in pixels, and the horizontal axis indicates the frame number.

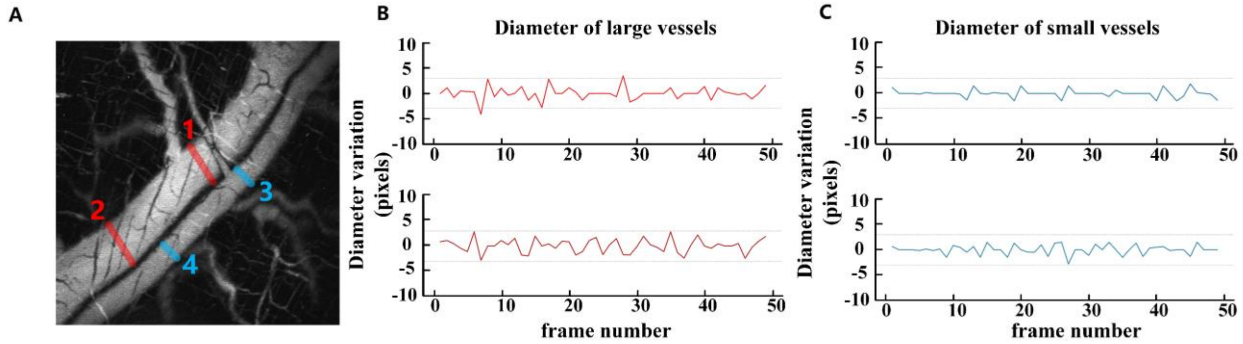

Fig. S8. Stability analysis of vessel diameter quantification under 10 Hz imaging. A, Maximum intensity projection images acquired at 10 Hz for quantitative analysis of motion-induced vessel diameter fluctuation. **B-C**, The diameter variation between consecutive frames at 10 Hz. For each frame, the vascular skeleton within the ROI is divided into three equal segments along the vessel centerline, and the centroid displacement of each segment between adjacent frames is calculated, the displacement between the centroids of adjacent frames can be converted into the change in diameter of the corresponding centroids of adjacent frames.

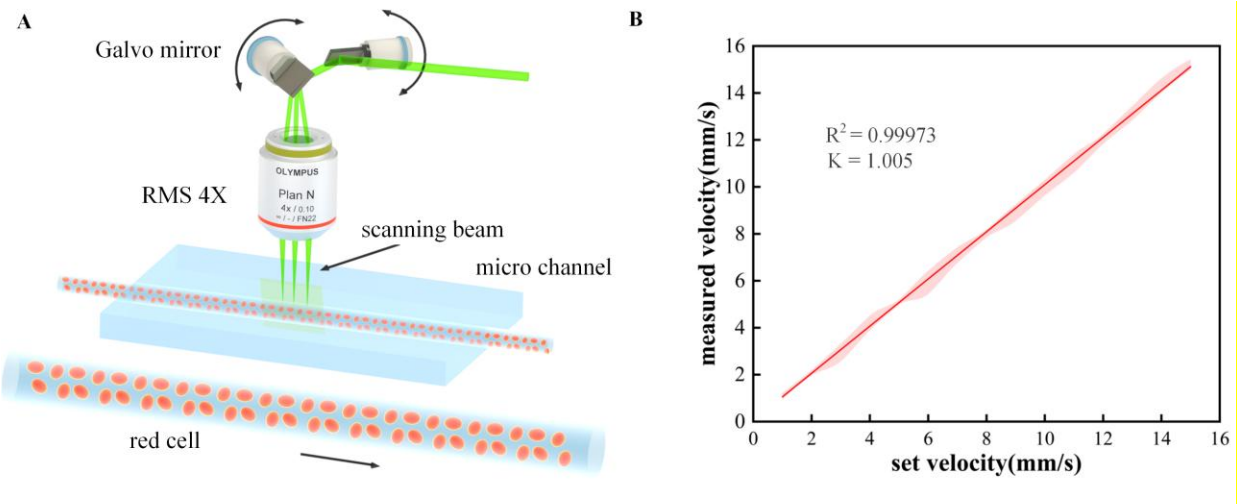

Fig. S9. Microfluidic blood flow velocity quantification experiment. **A**, Experiment setup. Anticoagulated whole blood collected from mouse was infused into a microfluidic channel (100 μm), and the flow velocity was precisely controlled over the range of 1–15 mm/s using a syringe pump (KD Scientific, LEGATO 210). **B**, Linear fitting showed excellent agreement between measured and preset velocities, with $R^2 = 0.99973$ and $K = 1.005$.

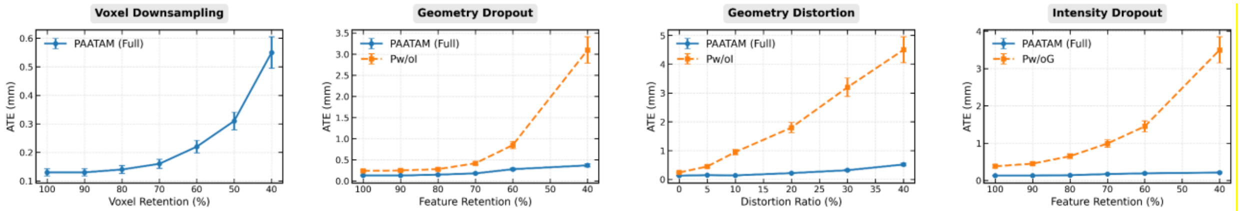

Fig. S10. Sensitivity analysis of mapping accuracy under synthetic data degradation. To evaluate robustness against extreme conditions like bleeding or tissue deformation, we retrospectively perturbed 5 simulated datasets with ground truth. Perturbations included random voxel downsampling, geometric/intensity feature dropout, and geometric distortion from 100% down to 40% retention/ratio. The ATE was quantified for full PAATAM and single-feature ablations (Pw/oI, Pw/oG). Results demonstrate that the hybrid feature coupling significantly extends the breakdown threshold. While single-feature tracking drifts into degeneracy below 70% feature retention, the hybrid coupling provides sufficient complementary constraints to prevent drift even at 40% retention.

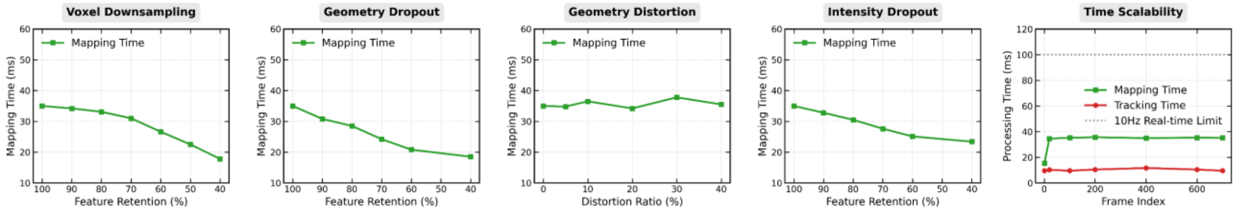

Fig. S11. Computational scalability and real-time performance analysis. Processing time across varying feature retention levels and long-term scanning sequences. The first four panels show mapping time correlating linearly with point cloud density (O(N) complexity), which is bounded by hardware sampling limits. The final panel displays tracking and mapping latency per frame over a 700-frame sequence (Phantom2 slow). The processing time forms a flat plateau well below the 100 ms (10 Hz) real-time limit. This confirms that sliding-window marginalization restricts backend factor-graph optimization to O(1) complexity, effectively preventing computational explosion during prolonged panoramic scanning.

Table S1. Collected Sequences details.

Type	Dataset	Scans	Distance(mm)	Max Speed
Simulated	Phantom2 slow	700	18.3	0.3
	Phantom2 median	350	18.2	0.5
	Phantom2 fast	175	17.0	1.0
	Phantom3 slow	109	6.9	1.6
	Phantom3 fast	112	14.2	3.3
Freehand	Phantom1	1048	N/A	N/A
	Mice Cecum1	801	N/A	N/A
	Mice Cecum2	780	N/A	N/A
	Mice Cross Organ	80	N/A	N/A
	Rat Stomach1	172	N/A	N/A
	Rat Stomach2	227	N/A	N/A
	Human Lip	326	N/A	N/A

Overview of collected sequences and their number of scans. The simulated scanning sequences are listed with distance and maximum movement speed.

Table S2. Ablation study on trajectory-related metrics across 5 simulated datasets.

	Trk	SM	BA	Pw/oG	Pw/oI	ATE [mm]			RTE [mm]		
						rmse	mean	std	rmse	mean	std
PAATAM	✓	-	-	-	-	0.3494	0.2892	0.1960	0.0284	0.0217	0.0182
	✓	✓	-	-	-	0.1796	0.1540	0.0924	0.0228	0.0190	0.0126
	✓	-	✓	-	-	0.3362	0.2910	0.1684	0.0350	0.0216	0.0275
	✓	✓	✓	-	-	0.1514	0.1313	0.0754	0.0229	0.0189	0.0129
	-	-	-	✓	-	0.4402	0.3826	0.2177	0.0534	0.0310	0.0435
	-	-	-	-	✓	0.3713	0.2405	0.2830	0.0617	0.0298	0.0541
LOAM	-	-	-	-	-	0.3763	0.2470	0.2839	0.0621	0.0295	0.0547
ICP	-	-	-	-	-	0.4351	0.3719	0.2259	0.0565	0.0286	0.0487
G-ICP	-	-	-	-	-	0.4353	0.3719	0.2261	0.0567	0.0287	0.0489
P2P-ICP	-	-	-	-	-	1.3200	1.0868	0.7492	0.0608	0.0298	0.0530

Trk: scan tracking; SM: scan to local map optimization; BA: local bundle adjustment; Pw/oG: PAATAM without geometric feature; Pw/oI: PAATAM without intensity feature.

Legends for movies S1 to S16

Movie S1. Accuracy test at slower speed movement in a simulated scan. The video shows 10Hz imaging (top left), real-time tracking and mapping with PAATAM (middle), and processes of other methods (right). A comparison of predicted trajectories with ground truth (bottom left) demonstrates PAATAM's global consistency over long sequences. The video is played at three times the speed.

Movie S2. Accuracy test at medium speed movement in a simulated scan. The video is played at double speed.

Movie S3. Accuracy test at higher speed movement in a simulated scan. A comparison of predicted trajectories with ground truth (bottom left) shows PAATAM's better accuracy under high sweep speed.

Movie S4. Simulated scan under various motions. The video shows 10Hz imaging (top left), real-time tracking and mapping with PAATAM (middle), and processes of other methods (right). A comparison of predicted trajectories with ground truth (bottom left) shows PAATAM's robustness under motion conditions.

Movie S5. Simulated scan under various motions. A comparison of predicted trajectories with ground truth (bottom left) shows PAATAM's robustness under intense motion conditions.

Movie S6. Visualization of iterative estimation. The process based solely on vascular geometric features (left) requires multiple iterations for convergence and may fail due to degeneracy. Estimation driven by vascular geometric and intensity features (right) converges faster and is more robust.

Movie S7. Handheld scanning in mice cecum1. The video shows 10 Hz handheld PAA imaging (top left), real-time tracking and mapping with PAATAM (middle), processes of other methods (right), and a comparison of predicted trajectories with ground truth (bottom left).

Movie S8. Display of mice cecum1 reconstruction result in 3D.

Movie S9. Handheld scanning in mice cecum2.

Movie S10. Display of mice cecum2 reconstruction result in 3D.

Movie S11. Handheld scanning in mice cross organ. The video shows a 10 Hz PAA sequence (left) and tracking and mapping with PAATAM (right).

Movie S12. Display of mice cross organ reconstruction result in 3D.

Movie S13. Handheld scanning in human lip.

Movie S14. Display of human lip reconstruction result in 3D.

Movie S15. Vascular panoramic mapping for partial gastrectomy. The first part shows real-time preoperative mapping under 10 Hz PAA scanning. The second part displays the surgical planning. The third part shows the remapping post-surgery. The fourth part demonstrates the postoperative assessment, achieving the expected preservation of blood vessels.

Movie S16. In vivo monitoring hemodynamic during left gastric vessel ligation in rat. After baseline gastric vascular data were acquired, the left gastric artery and vein were ligated with a hemostatic clamp, which was released after 40 seconds to collect blood flow data over the entire process
